# Treatment Extension of Pegylated Interferon Alpha and Ribavirin Does Not Improve SVR in Patients with Genotypes 2/3 without Rapid Virological Response (OPTEX Trial): A Prospective, Randomized, Two-Arm, Multicentre Phase IV Clinical Trial

**DOI:** 10.1371/journal.pone.0128069

**Published:** 2015-06-09

**Authors:** Benjamin Heidrich, Hans-Jörg Cordes, Hartwig Klinker, Bernd Möller, Uwe Naumann, Martin Rössle, Michael R. Kraus, Klaus H. Böker, Christoph Roggel, Marcus Schuchmann, Albrecht Stoehr, Andreas Trein, Svenja Hardtke, Andrea Gonnermann, Armin Koch, Heiner Wedemeyer, Michael P. Manns, Markus Cornberg

**Affiliations:** 1 Department of Gastroenterology, Hepatology and Endocrinology, Hannover Medical School, Hannover, Germany; 2 HepNet Study-House, German Liver Foundation, Hannover, Germany; 3 German Center for Infection Research (DZIF), partner site Hannover-Braunschweig, Germany; 4 Gastroenterological Practice, Frankfurt, Germany; 5 Department of Internal Medicine II, University of Würzburg, Würzburg, Germany; 6 Medical Practice, Leberzentrum, Berlin, Germany; 7 Center for Addiction-Medicine, Hepatology and HIV, Praxiszentrum Kaiserdamm, Berlin, Germany; 8 Praxiszentrum Gastroenterologie, Freiburg, Germany; 9 Department of Internal Medicine, Hospital Altötting-Burghausen, Germany; 10 Medical Practice, Hannover, Germany; 11 Medical Practice, Minden, Germany; 12 Johannes Gutenberg University Mainz, Mainz, Germany; 13 IFI Institute for Interdisciplinary Medicine, Asklepios Klinik St Georg, Hamburg, Germany; 14 Medical Practice, Stuttgart, Germany; 15 Institute of Biostatistics, Hannover Medical School, Hannover, Germany; Taipei Veterans General Hosptial, TAIWAN

## Abstract

Although sofosbuvir has been approved for patients with genotypes 2/3 (G2/3), many parts of the world still consider pegylated Interferon alpha (P) and ribavirin (R) as standard of care for G2/3. Patients with rapid virological response (RVR) show response rates >80%. However, SVR (sustained virological response) in non-RVR patients is not satisfactory. Longer treatment duration may be required but evidence from prospective trials are lacking. A total of 1006 chronic HCV genotype 2/3 patients treated with P/R were recruited into a German HepNet multicenter screening registry. Of those, only 226 patients were still HCV RNA positive at week 4 (non-RVR). Non-RVR patients with ongoing response after 24 weeks P-2b/R qualified for OPTEX, a randomized trial investigating treatment extension of additional 24 weeks (total 48 weeks, Group A) or additional 12 weeks (total 36 weeks, group B) of 1.5 μg/kg P-2b and 800-1400 mg R. Due to the low number of patients without RVR, the number of 150 anticipated study patients was not met and only 99 non-RVR patients (n=50 Group A, n=49 Group B) could be enrolled into the OPTEX trial. Baseline factors did not differ between groups. Sixteen patients had G2 and 83 patients G3. Based on the ITT (intention-to-treat) analysis, 68% [55%; 81%] in Group A and 57% [43%; 71%] in Group B achieved SVR (p= 0.31). The primary endpoint of better SVR rates in Group A compared to a historical control group (SVR 70%) was not met. In conclusion, approximately 23% of G2/3 patients did not achieve RVR in a real world setting. However, subsequent recruitment in a treatment-extension study was difficult. Prolonged therapy beyond 24 weeks did not result in higher SVR compared to a historical control group.

**Trial Registration:**

ClinicalTrials.gov NCT00803309

## Introduction

World-wide 64–103 million people are considered to be chronically infected with the hepatitis C virus (HCV) [1]. Despite the approval of potent drugs the incidence of liver transplantations, decompensated liver cirrhosis and hepatocellular carcinoma (HCC) will further increase [2]. In 2011, boceprevir and telaprevir, first generation protease inhibitors have been approved for the treatment of HCV genotype 1 in combination with pegylated interferon alpha (PEG-IFN) and ribavirin. Since the first approval of direct acting antivirals (DAA) in 2011 more compounds have been discovered. DAA target the NS3/4A protease, NS5B polymerase and the NS5A replication complex [3,4]. In 2014, the treatment of genotype 2 and 3 patients dramatically changed due to the approval of sofosbuvir a new NS5B polymerase inhibitor with pangenotypic efficacy. In genotype 2 and 3 patients interferon-free therapy is already possible and approved [5–7]. Current guidelines and expert recommendations released recommendations that patients with genotype 2 should be treated for 12 weeks with sofosbuvir and ribavirin whereas genotype 3 should be treated with triple therapy (sofosbuvir, pegylated Interferon alpha and ribavirin) for 12 weeks or with sofosbuvir and ribavirin for 24 weeks [8,9].

Before the approval of sofosbuvir in Western countries and still in developing countries with low financial resources and problems to reimburse sofosbuvir, patients with HCV genotype 2 and 3, especially those with genotype 3 and unfavorable predictors of response remained a challenge in the treatment of chronic hepatitis C [10,11]. Patients treated with standard of care consisting of pegylated interferon alpha and ribavirin with rapid virological response (RVR) show response rates >80% even with shorter than 24 weeks of treatment duration [12–18]. However, sustained virological response (SVR) in non-RVR patients is not satisfactory especially in patients with genotype 3. Longer treatment durations based on PEG-IFN and ribavirin were considered as strategy to improve SVR rates in patients with non-RVR before the approval of DAA like sofosbuvir. However, evidence from prospective trials investigating the effect of therapy prolongation with PEG-IFN and ribavirin are sparse [10,19]. The primary objective of OPTEX (NCT00803309) was to compare the efficacy of treatment duration of 36–48 weeks (treatment extension of 12–24 weeks) with a historical control group treated for 24 weeks in non-RVR patients with HCV genotype 2/3 who were treated with standard pegylated interferon alpha-2b and ribavirin.

## Material and Methods

### Study design

**Fig 1:**
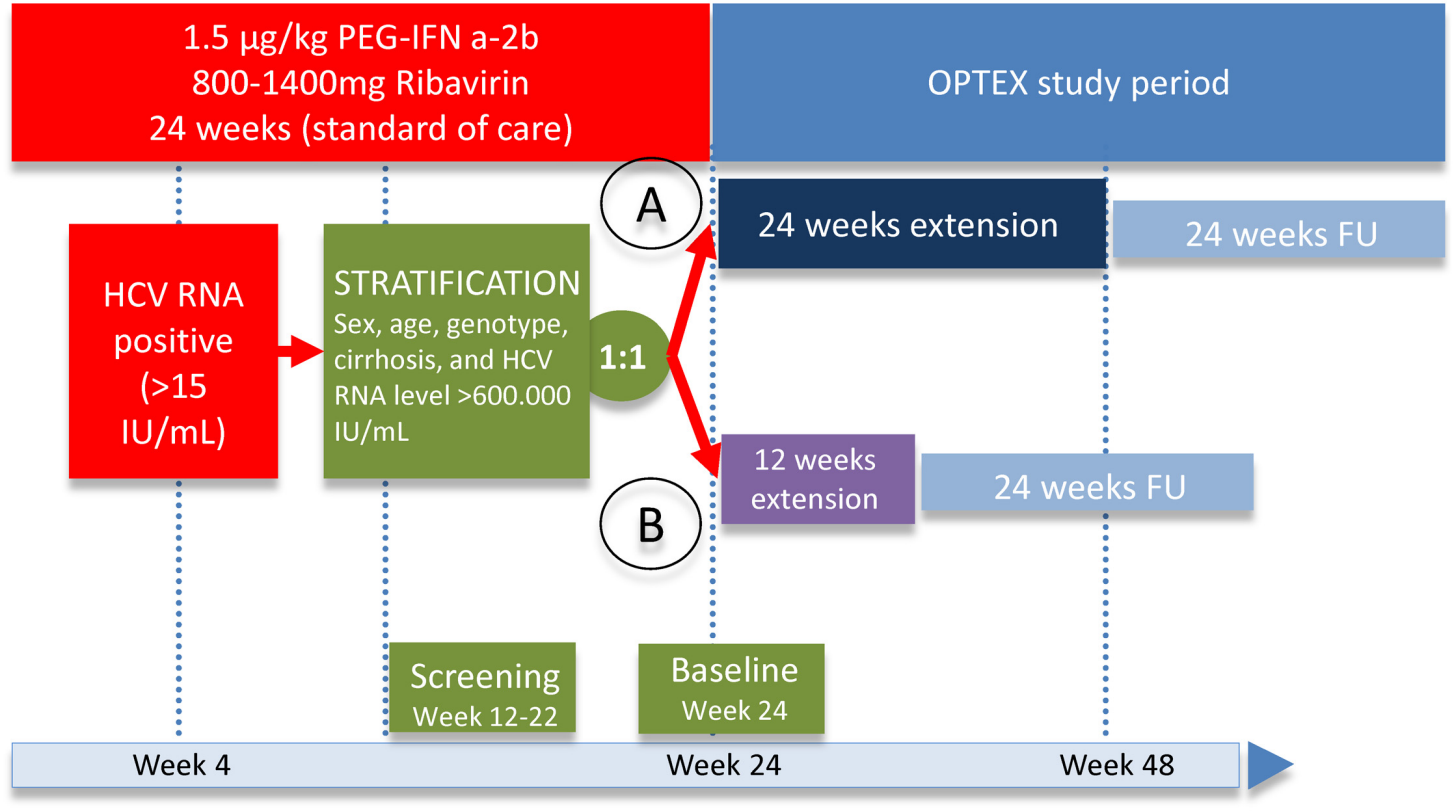
Study design of the OPTEX 2/3 trial.

This study was a prospective, two-arm, multicentre phase IV clinical trial examining the efficacy of treatment prolongation of additional 24 weeks (group A, total treatment duration 48 weeks) or additional 12 weeks (group B, total treatment duration 36 weeks) with 1.5 μg/kg PEG-IFN alpha-2b and 800–1400 mg/day ribavirin in HCV infected patients with genotype 2 or 3 and no rapid virological response (HCV RNA-positive at week 4) compared to standard treatment duration (historical control group). At the beginning all patients were treated with 1.5 μg/kg PEG-IFN alpha-2b and 800–1400 mg/day ribavirin for 24 weeks. Patients without RVR at week 4 and EVR (> 2 log decline of HCV RNA at week 12 of treatment) were eligible for this study. The screening period was between week 12 and 22 of standard of care therapy. Immediately after the end of treatment patients were treated for additional 12 or 24 weeks depending on the study arm (Fig 1).

### Study objectives

The primary objective of this study was reduction of relapse rate 24 weeks after the end of treatment and thus improved sustained virological response (SVR24) in the group with a prolongation of 24 weeks (group A) in comparison with SVR24 rates in patients without treatment prolongation (historical control group with SVR-rate 70%) [13,18]. Key secondary analyses were the comparison of Group B against the historical control group and the comparison of Group A versus Group B regarding the SVR rates. Patients were randomized in a 1:1 ratio to Group A and B. Randomization was stratified by sex, age, genotype, cirrhosis, and HCV RNA level >600.000IU/ml.

### Control group

In this study, a historical control group was used instead of an additional arm with standard of care for 24 weeks because the expected number of patients eligible for this study was too low. In the trials of Zeuzem et al. and Dalgard et al. Caucasian patients with HCV genotype 2 or 3 were treated with a similar treatment regimen (1.5 μg/kg PEG-IFN alpha-2b plus 800–1400mg RBV for 24 weeks). Patients without RVR but EVR (early virological response) achieved SVR rates from 56% to 75%. We considered an SVR rate of 70% as historical control which is at the upper end because our cohort does not include the null-responder at week 12 of treatment (Table 1) [13,18].

**Table 1:** Historical control group.

Study	Treatment regimen	Patients without RVR	SVR without RVR
Zeuzem et al., [18]	1.5 μg/kg 24 weeks PEG-IFN alpha-2b plus 800–1400mg RBV (24weeks)	HCV GT 2 n = 0HCV GT 3 n = 35	n.a.24/35* (68.6%)
Dalgard et al., [13]	1.5 μg/kg 24 weeks PEG-IFN alpha-2b plus 800–1400mg RBV (24weeks)	HCV GT 2 n = 20HCV GT 3 n = 96	15/20** (75%)54/96** (56.3%)
**Both studies**	1.5 μg/kg 24 weeks PEG-IFN alpha-2b plus 800–1400mg RBV (24weeks)	HCV GT 2/3 n = 151	93/151 (61.8%)

* Patients who were HCV-RNA positive (>29 IU/mL) at week 4 but HCV-RNA negative at week 12

** Patients who were HCV-RNA positive (>50 IU/mL) at week 4 but HCV-RNA negative at week 12

### Study population

A total sample size 150 patients was initially planned, 75 patients in each group. Patients at the age of at least 18 years who fulfilled the in- and exclusion criteria were eligible for this study. Patients for the study were recruited from a prospective national multicentre centre registry. For detailed in- and exclusion criteria see supporting materials. First patient screened was on 18^th^ December 2008, first start of study therapy and first baseline visit was on 30^th^ January 2009. The recruitment of patients with non-RVR was slower than calculated and based on the new developments in treatment of chronic hepatitis C in Germany it was decided to stop the recruitment on 22^nd^ October 2012. The last visit in study of the last enrolled patient was on 29^th^ July 2013.

### Definition of liver cirrhosis

Diagnosis of cirrhosis was either based on liver histology or non-invasive methods such as ultrasound, FibroScan or biochemical results. Liver cirrhosis in biopsies was defined as F4 in Metavir or F5-6 in ISHAK. In addition, diagnosis of liver cirrhosis was based on ultrasound results assessed by the local physician. Liver stiffness ≥12.5 kPa was considered as cirrhosis [16]. Patients with at least two of the following criteria platelets <100/nL, AST/ALT ratio >1, bilirubin >1.5 ULN and albumin <35 g/L fulfilled biochemical assessment of cirrhosis. Individuals were considered having cirrhosis if one of the definitions above was imbued [15].

### Ethical approval

Ethics committee at each participating institution approved the interventional trial of the German Competence Network for Viral Hepatitis (Hep-Net) and each patient signed a written informed consent form. The study has been performed according to the World Medical Association Declaration of Helsinki (http://www.wma.net/e/policy/b3.htm). The ethics committee of the Hannover medical school was the leading ethics committee and approved the procedures (Vote No. 3860). The study procedures are in line with German law. The following ethics committees of the federal states approved the protocol: Medical association of Schleswig-Holstein, Berlin, Bremen, Saxony, North Rhine, Westphalia-Lippe, Bavaria, Hessen, Hamburg, Lower Saxony, Saarland, Baden-Wuerttemberg, Rhineland-Palatinate, and Saxony-Anhalt. Additionally, the following local ethics committees of universities and medical faculties approved the study protocol: Aachen, Bonn, Duisburg-Essen, Frankfurt, Freiburg, Heidelberg, Jena, Leipzig, Lübeck, Würzburg, Ulm, Tübingen, Münster, Regensburg, Magdeburg, Mannheim, Heidelberg, and Munich.

The ethical committee of Hannover Medical School approved IL28B genotyping of patients within the HepNet genotype 2/3 registry on August 10^th^ 2010. IL28B genotyping was not part of the OPTEX trial and patients gave independent informed consent for IL28B testing.

### Study registration

The official title of the study is “Optimization of Treatment for Patients With Chronic Hepatitis C Infected With HCV-genotype 2 or 3: 12 vs. 24 Weeks of Treatment Extension for Patients Without Rapid Virological Response” (NCT00803309) (https://clinicaltrials.gov/ct2/show/NCT00803309?term=optex&rank=1).

### Statistical analysis

The primary objective of this trial was an improved SVR-rate in Group A compared to the SVR of the historical control group (70%). For the primary analysis SVR rate of Group A was calculated with 95% Wald confidence intervals (CI). The study was considered successful, if the lower bound of the 95% Wald CI of the SVR rate of Group A is above 70%. As key secondary analysis the SVR rate of Group B (12 week prolongation) was compared to the SVR rate of the historical control group [13,18]. The analysis was carried out in line with the primary analysis. Another key secondary objective was to compare Group A with Group B. For this comparison the analysis was adjusted for the stratification variables and therefore Mantel-Haenszel risk differences were used for the comparison of these two groups. Due to too many stratification variables and the reduced sample size, empty cells were determined in the subgroups. Therefore, stratification variables had to be removed for the analysis. The order of the stratification variables was age, sex, genotype, cirrhosis and HCV-RNA-level. According to the pre-specified order, the last stratification variable was excluded until calculation was possible and the analysis was finally adjusted for age and gender.

The primary analysis was conducted according to the intention-to-treat (ITT) principle in all randomized patients. Patients were asked to participate in the study during the standard therapy between week 12 and 22 and some patients dropped before they had started the study. Sensitivity analysis was conducted on all patients that had a baseline visit and therefore started the study therapy (modified ITT; mITT). Additionally, a dataset with only compliant patients was created, which was defined as by having completed the study according to the protocol and having received at least 80% of the interferon and ribavirin doses for at least 80% of the planned treatment duration (completers analyses). All statistical analyses were conducted using SAS (Version 9.3).

Two strategies to replace missing values on HCV RNA (qualitative) were used: For those patients that had missing values on HCV RNA during the course of the study, but were HCV RNA-negative at the previous and the next visit, HCV RNA was considered to be negative and missing values were re-placed with negative. All other missing values were replaced as being positive, following a conservative strategy for the primary analysis (ITT-principle). Missing values in key secondary laboratory variables were replaced with last observation carried forward (LOCF). Missing values for quantitative HCV RNA were not replaced as this was not a pre-specified key secondary variable and LOCF methods may lead to an anti-conservative estimation if patients had a relapse.

Quantitative variables are given as mean, min, max and standard deviation (SD). Group A and Group B were compared exploratory with t-tests for independent groups. For laboratory data, the t-test was calculated after logarithmic transformation and estimates were back transformed thereafter to properly reflect the skewed distribution. For categorical variables absolute (and relative) frequencies are presented and compared using χ^2^-test and Fisher’s exact test respectively. Secondary comparisons were assessed using a two-sided significance level of 5%. As sensitivity analysis a multivariate logistic regression analysis was performed with all stratification variables plus IL28B genotypes.

### HCV RNA quantification and laboratory testing

The assessment of quantitative HCV RNA was done at baseline, week 12 and week 24 as well as at every follow-up visit (FU4, FU12 and FU24). All samples were tested centrally for HCV RNA in a lab at Hannover Medical School. The Cobas-TaqMan assay with a lower limit of quantification of 15 IU/mL was used for quantification of HCV RNA. The assay was used according to the specific manufacturer’s instructions. All laboratory testing was performed locally at each site. Each investigator was responsible to submit appropriate laboratory certificates and all ranges of normal values to the HEP-NET study coordinator. Only laboratories were involved taking part in quality control programs regularly.

### IL28B rs12979860 genotyping

IL28B single-nucleotide polymorphism rs12979860 genotyping was performed using real-time polymerase chain reaction and melting curve analysis in the Light Cycler 480 II System (Roche, Mannheim, Germany). DNA was extracted from EDTA-blood samples using the DNeasy purification Kit (QIAGEN, Hilden, Germany). Primers and hybridization probes were purchased from TIB MOLBIOL (Berlin, Germany).

### Efficacy Results on Analysis of quality of life

The SF-36 Mental and Physical Summary Scores were standardized to a mean of 50 and a standard deviation of 10 according to the U.S. general population. Differences between German and U.S. general population are only minimal [20]. Choosing the American population gives internationally comparable results. Questionnaire SF-36 was planned to be assessed at baseline, at treatment week 12 and 24 (only group A) during study as well as at follow-up 12 and 24.

## Results

### Patient population

**Fig 2:**
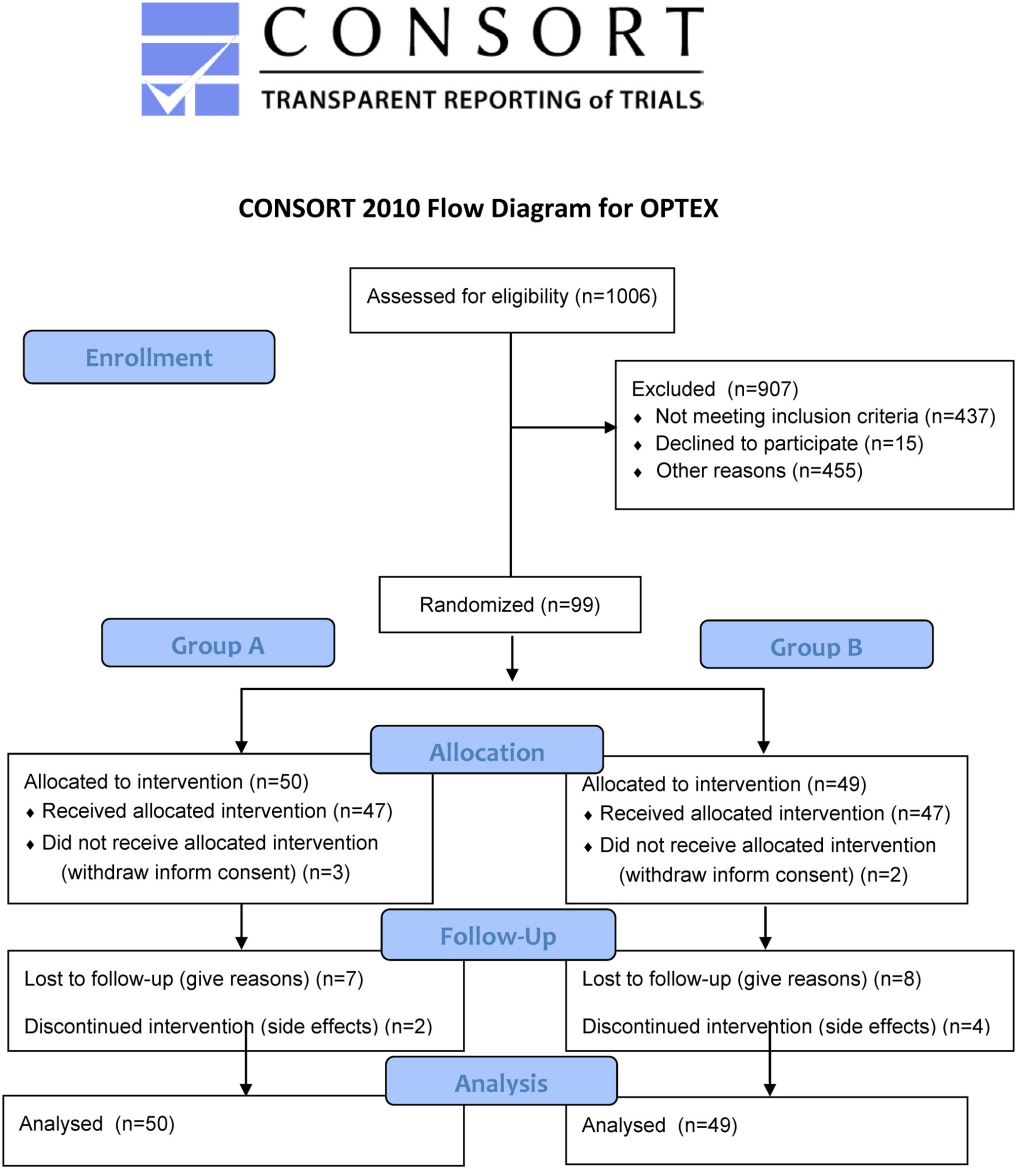
CONSORT flowchart for the OPTEX trial.

**Fig 3:**
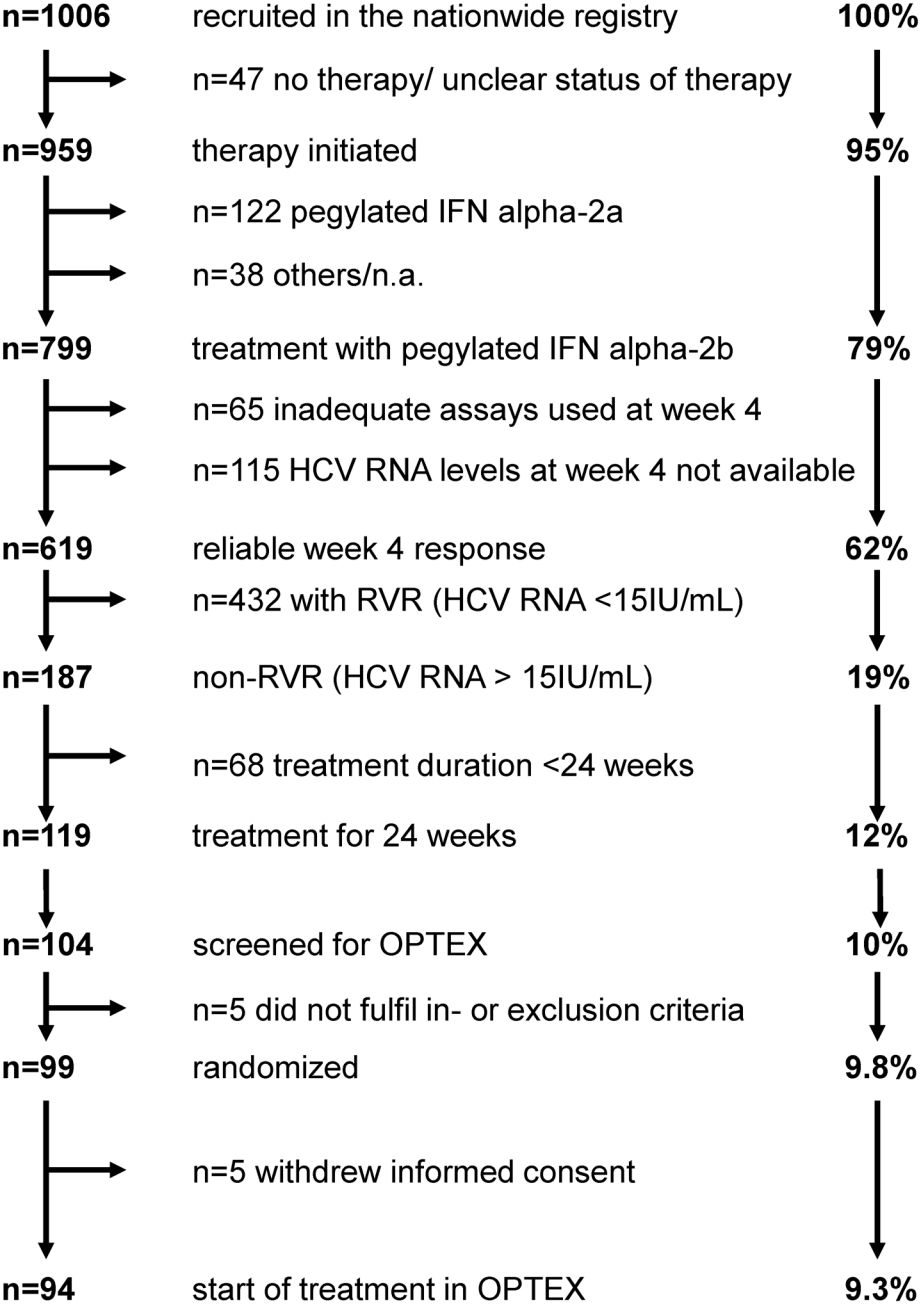
Flow chart of patients recruited for the OPTEX trial.

In total, 1006 patients were included in a nationwide registry for patients with hepatitis C genotype 2 or 3 [15]. Out of these, 226 tested HCV RNA-positive at week 4 (non-RVR). In total, 104 out of the 1006 (10%) patients from the nationwide registry could be screened between week 12 and 22 of standard therapy for this study. Ninety-nine (9.8%) patients fulfilled in- and exclusion criteria, of which 83 patients had genotype 3 (n = 67 genotype 3a, n = 16 subtype n.a.) and 16 patients had genotype 2 (n = 4 genotype 2a, n = 6 genotype 2b, n = 6 n.a.). 50 patients were randomized into Group A and 49 patients into Group B. Patients were randomized during the screening phase (standard treatment week 12–22) and 5 patients dropped out before start of the study (week 24 of ongoing treatment) but after randomization (three patients in Group A and two in Group B). For further analysis all randomized patients were included. The mean age of patients at baseline was 45.0 ± 8.9 years with the majority of patients being male. We observed no statistical significant differences in baseline characteristics between both groups with exception in bilirubin. Patients in Group A had significantly higher bilirubin levels compared to Group B (0.65 ± 0.33 vs. 0.51 ± 0.23; p = 0.016). However, in only few of the patients bilirubin levels were above the upper limit of normal (Table 2). The patient flow is visualized in Figs 2 and 3.

**Table 2:** Baseline characteristics of all randomized patients at study initiation.

	Group A (24 weeks) n = 50	Group B (12 weeks) n = 49	p-value	Dalgard et al. [13]n = 130 (SVR data in n = 116)
**Age [years]**			0.7	
Mean ± SDRangen =	44.7 ± 8.525–6050	45.3 ± 9.422–6349		4322–61
**Sex [n = (%)]**			0.7	
Male	32 (64%)	33 (67%)		76 (59%)
**Genotype [n = (%)]**			0.6	
Genotype 2Genotype 3	9 (18%)41 (82%)	7 (14%)42 (85%)		25 (19%)105 (81%)
**HCV RNA BL ≥ 600.000 IU/mL [n = (%)]**			0.9	
	29 (58%)	28 (57%)		97 (75%)*
**APRI score**			0.6	
Mean ± SDRangen =	1.24 ± 3.060.22–20.9446	0.99 ± 1.590.11–9.5243		
**APRI score [n = (%)]**			1.0	
<2>2Not available	41 (82%)5 (10%)4 (8%)	39 (80%)4 (8%)6 (12%)		98 (75%)26 (20%)6 (5%)
**SVR rates according to IL28B-rs8099960 [SVR/total (%)]**				
MissingCCCTTT	13/19 (68%)2/6 (33%)15/20 (75%)4/5 (80%)	9/15 (60%)6/11 (55%)10/17 (59%)3/6 (50%)		CC vs. non-CCp = 0.153
**BMI [kg/m^2^]**			0.2	
Mean ± SDRangen =	26.0 ± 5.519.6–42.950	24.7 ± 3.919.0–34.849		
**Cirrhosis [n = (%)]**			1.0	
	6 (12%)	6 (12%)		
**Steatosis [n = (%)]**			0.5	
	10 (25%)	9 (20%)		
**AST [U/L]**			0.5	
Mean ± SDRangen =	51.5 ± 78.019–51346	42.1 ± 35.513–17944		
**ALT [U/L]**			0.5	
Mean ± SDRangen =	46.0 ± 58.211–30347	39.2 ± 40.88–22547		
**Bilirubin [mg/dl]**			0.016	
Mean ± SDRangen =	0.7 ± 0.30.3–1.947	0.5 ± 0.20.1–1.147		
**Creatinine [mg/dl]**			0.5	
	0.8 ± 0.20.6–1.346	0.8 ± 0.10.5–1.147		
**Leucocytes [10/μl]**			0.5	
Mean ± SDRangen =	3.5 ± 1.82.0–11.447	3.7 ± 1.61.9–8.346		
**Platelets [10/μl]**			0.3	
Mean ± SDRangen =	153.1 ± 49.044–24446	167.35 ± 76.736–16246		

* In Delgard et al., 400.000IU/mL was used as cut-off for high and low viral load

### Adverse events

All patients in Group A and B, respectively, received at least one dose of study medication. In total, 74 (75%) individuals completed the study according to the study protocol (39 (78%) individuals in Group A and 35 (71%) in Group B, respectively). One patient in Group A died during the study due to hepatocellular carcinoma. In addition, six patients withdrew informed consent (three in each group) without mentioning the reason, nine patients were lost to follow-up (three in Group A and six in Group B), four patients stopped the study prematurely due to therapy failure (one in Group A and three in Group B) and five patients did not finish the study according protocol due to other reasons.

Overall, 302 adverse events were observed with 191 events in Group A and 111 in Group B. At least one adverse event was observed in 80 out of 99 (89%) randomized patients and in 79 out of 94 (84%) patients who received at least one dose of medication. In Group A 42 and in Group B 38 patients suffered from at least one adverse event. The adverse events were those typically associated with PEG-IFN or ribavirin treatment, and no unexpected adverse events occurred. At EOT skin and subcutaneous disorders were the group of adverse events with the highest frequency in both groups with 56% in Group A and 41% in Group B (p = 0.13) followed by psychiatric disorders in 48% and 25%, respectively (p = 0.02). The third common adverse events were general disorders and administration site conditions with 42% in Group A and 29% in Group B (p = 0.16). The nine serious adverse events that occurred in the study are described in detail in table 3. Dose modifications were done only in the minority of patients without being statistical significant between both treatment groups. In Group A and B the dose of PEG-IFN was increased in one and two patients, respectively. Dose reduction was necessary in four patients in Group A and in three patients in Group B. The dose of RBV was increased in one patient out of Group B and decreased in two patients in each treatment group.

**Table 3:** Severe adverse events.

SAE No.	Sex	Age	Arm	SAE term	Patient No.	Causality	Outcome
1	F	41	B	Morbus meniere, anemia, dehydration (exsiccosis)	17	Anemia probably related;Exsiccosis possibly related	Study medication stopped, anemia resolved
2	F	39	B	Biliary pancreatitis due to gallstones	6	Not related	Pancreatitis resolved, gallstones removed
2	F	40	B	Gastric lymphoma	6	Not related	SAE occurred during FU; study drug already terminated
3	M	46	B	Intracerebral bleeding, epilepsy	35	Not related	Patient was no longer receiving medication
4	M	42	B	Effusion of pericard and pleura	58	Possible	Study medication terminated
5	M	31	A	Pregnancy of partner	73	Not related	Patient was informed about birth control
6	M	40	A	Epigastralgia	76	Not related	Stent placement
7	M	40	A	Pyrexia, cholecystisis	76	Possible	Was no longer receiving treatment
8	M	40	A	Epigastralgia	76	Possible	Ribavirin discontinued for 6 days
9	M	40	A	Death	76	Not related	Death due to HCC

### Treatment efficacy

The primary end point, reduction of relapse rate (HCV-RNA positive in serum by a standard HCV-PCR with a detection limit of at least 15 IU/ml) 24 weeks after the end of treatment and thus improvement of sustained virological response rates (SVR) in patients without RVR and 48 weeks of therapy (Group A) was achieved by 34 out of 50 (68%) patients which is similar to the historical control group with SVR of 70% (p = 0.6191). Therefore, the primary aim of this study was not achieved.

**Fig 4:**
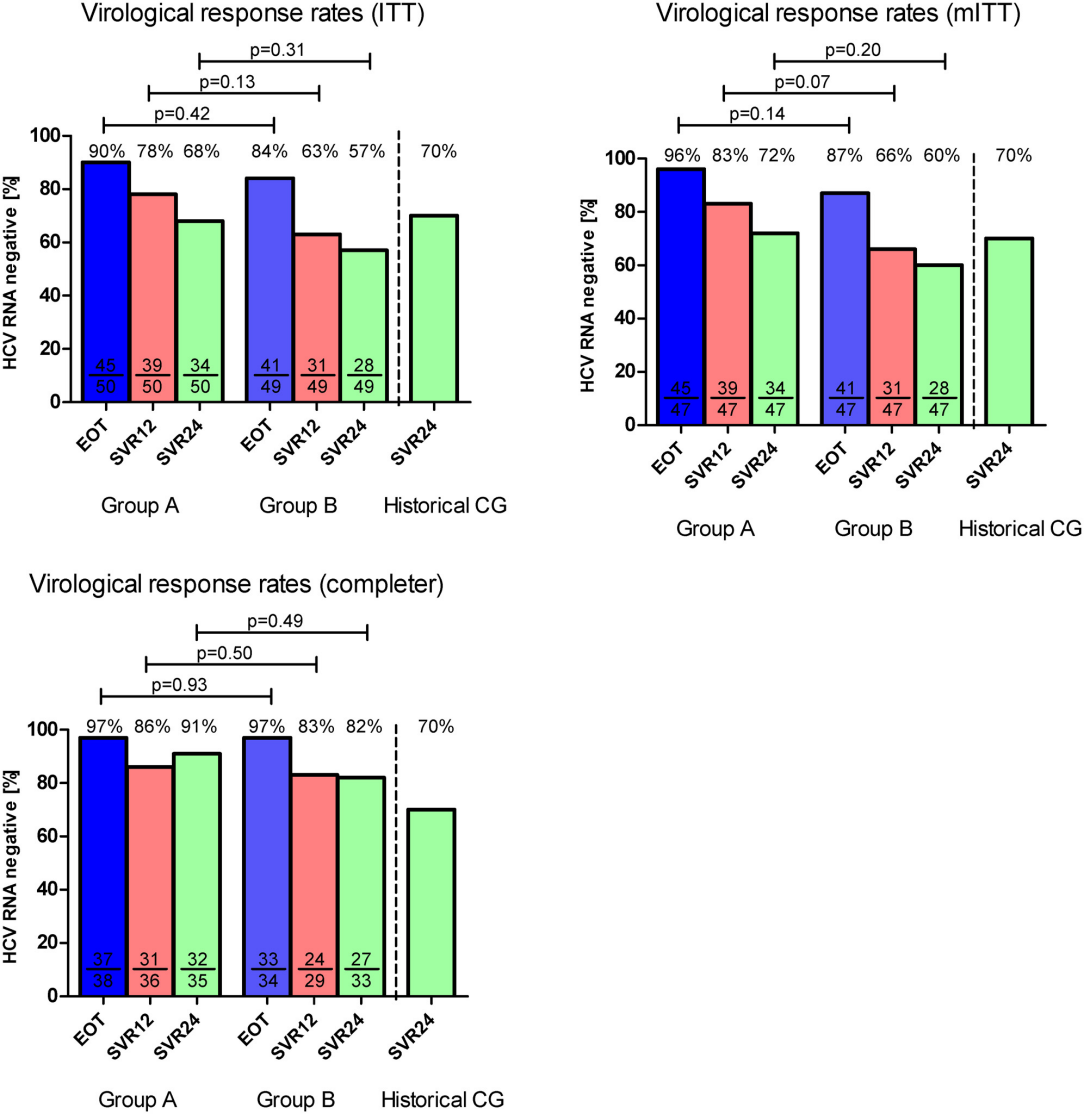
Virological response rates at EOT, FU12 and FU24 in the ITT, mITT and completer population.

**Fig 5:**
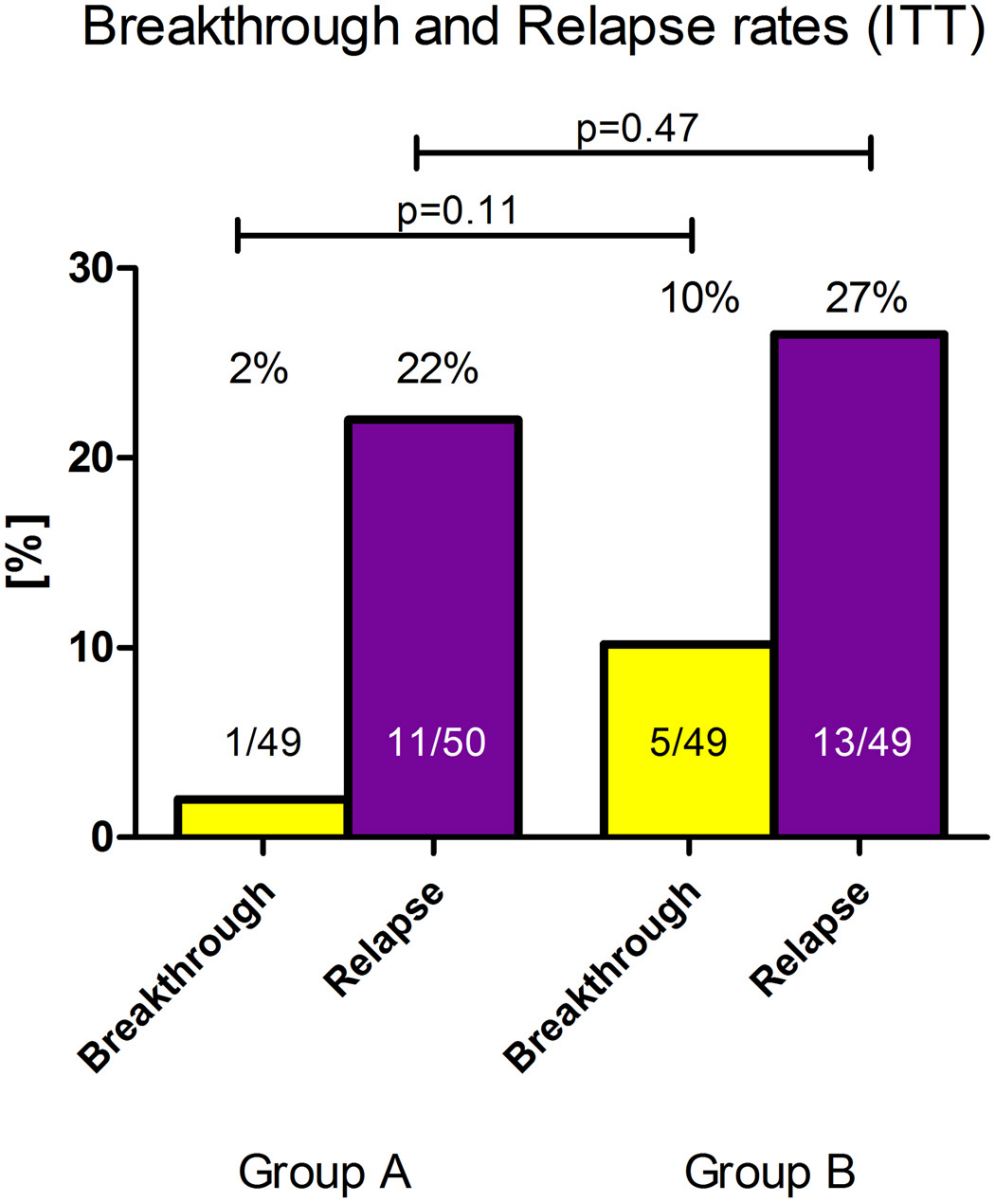
Virological breakthrough and relapse rates in treatment Group A and B in different populations.

No statistical significant differences regarding virological response rates were observed between Group A and Group B (Fig 4). in the ITT analysis as well as in the analysis of the completer only (Table 4, Subgroups in Table 5). In Group B no significant differences to the control group were observed at FU24. The breakthrough and relapse rates were comparable in both treatment groups in all populations (completer breakthrough: 3% vs. 0%; p = 1.0 and relapse: 13% vs. 18%; p = 0.86) (Fig 5). Breakthrough was defined as reappearance of HCV RNA during therapy.

**Table 4:** Virological response rates.

	Timepoint	Frequency (%)	95%-CI	p-value*
**Group A**	**EOT**	**45/50(90.00%)**	**[81.68%;98.32%]**	
	**FU12**	**39/50(78.00%)**	**[66.52%;89.48%]**	
	**FU24**	**34/50(68.00%)**	**[55.07%;80.93%]**	**0.6191**
**Group B**	**EOT**	**41/49(83.67%)**	**[73.32%;94.02%]**	
	**FU12**	**31/49(63.27%)**	**[49.77%;76.76%]**	
	**FU24**	**28/49(57.14%)**	**[43.29%;71.00%]**	**0.9655**

* One-sided p-values (α = 0.025); comparison to the historical control group

**Table 5:** Subgroupanalysis.

ITT population	SVR24-rate				
Stratum		Group A	Group B	RD (95%CI)	p-value
Age	<40 years	12/16 (75.00%)	8/12(66.67%)	8.33% (-25.75%;42.41%)	0.6318
	>40 years	22/34 (64.71%)	20/37(54.05%)	10.65% (-12.06%;33.36%)	0.3580
Gender	Male	18/32(56.25%)	17/33(51.25%)	4.73%(-19.48%;28.95%)	0.7015
	Female	16/18(88.89%)	11/16(68.75%)	20.14%(-6.82;47.09%)	0.1431
Genotype	2	7/9(77.78%)	5/7(71.43%)	6.35%(-36.75%;49.45%)	0.7728
	3	27/41(65.85%)	23/42(54.76%)	11.09%(-9.82;32.00%)	0.2985
Cirrhosis	Negativ	32/44(72.73%)	27/43(62.79%)	9.94%(-9.61%;29.48%)	0.3190
	Positiv	2/6(33.33%)	1/6(16.67%)	16.67%(-31.42%;64.75%)	0.4969
HCV RNA	>600.000 IU/ml	21/29(72.41%)	17/28(60.71%)	11.70%(-12.63%;36.03%)	0.3459
	<600.000 IU/ml	13/21(61.90%)	11/21(52.38%)	9.52%(-20.27%;39.32%)	0.5310

A multivariate logistic regression model was performed to analyse which factor was associated with SVR. From all factors that were used for stratification plus IL28B-rs12979860, female sex was associated with SVR. IL28B-rs12979860 genotype did not influence SVR (Table 6).

**Table 6:** Multivariate logistic regression model.

Odds Ratio Estimates				
Effect	Point Estimate	95% Wald Confidence Limits		Pr > ChiSq
Group A vs Group B	1.019	0.274	3.793	0.9774
Age <40 years vs >40 years	0.689	0.136	3.498	0.6527
male vs female	0.109	0.019	0.637	0.0139
Genotype 2 vs Genotype 3	5.066	0.772	33.258	0.0910
Liver cirrhosis negativ vs positiv	3.970	0.546	28.847	0.1731
HCV RNA >600000 IU/mL vs <600000 IU/mL	1.658	0.411	6.693	0.4776
IL28B rs12979860 CC vs TT or CT	0.139	0.017	1.103	0.0619

A secondary endpoint was biochemical response at end of treatment and 24 weeks after therapy cessation. Response was defined as ALT and/ or AST normalization (≤ 1.5x upper limit of normal) at that specific time point. Overall, the majority of patients achieved a biochemical response within the ITT and in the group of completer. In all specific patient populations we observed no statistical significant differences between Group A and B at FU24. In Group A 92% of completer achieved normalization of ALT and 88% in Group B

**Fig 6:**
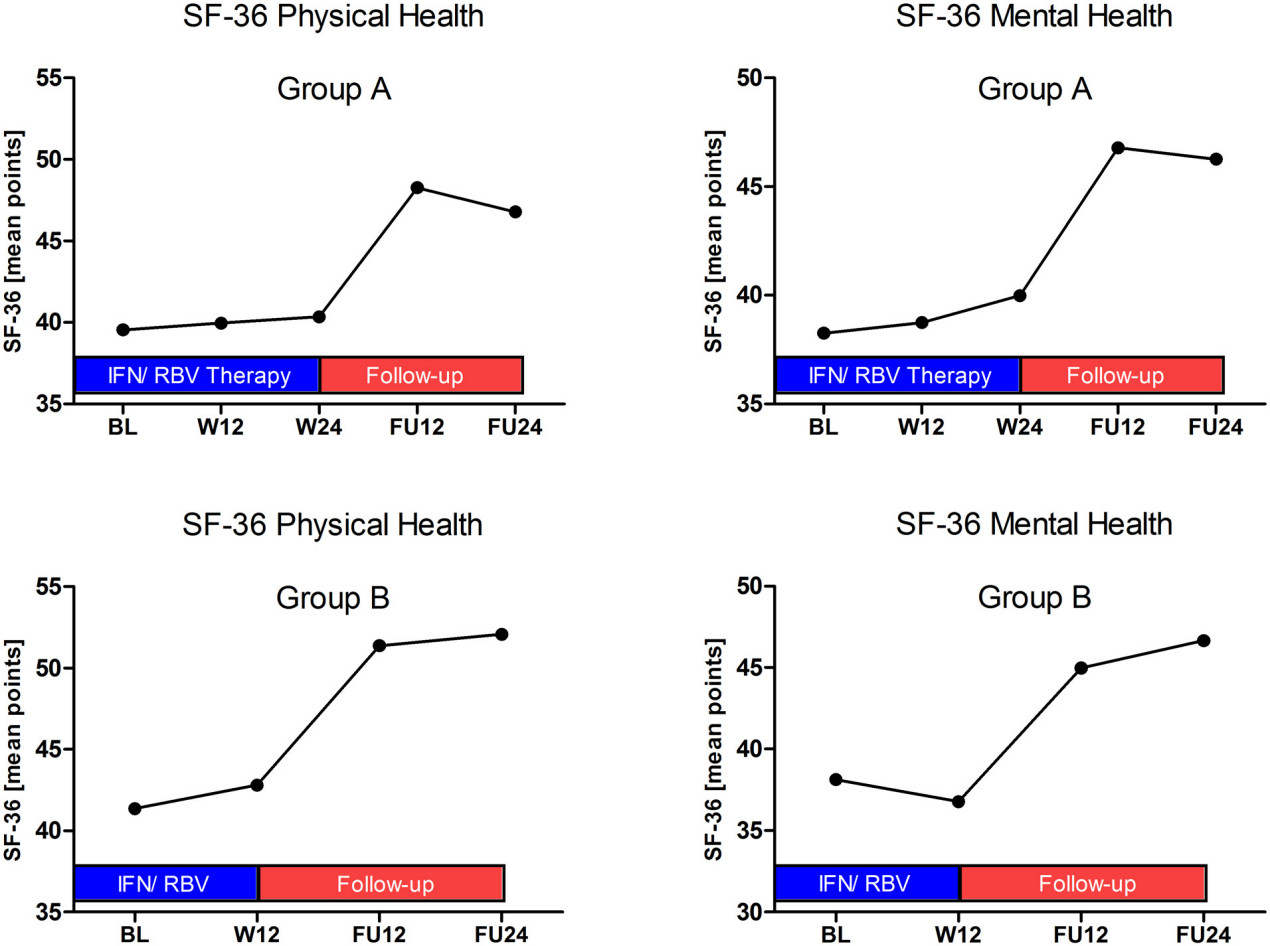
Results of SF-36 questionnaires analysing quality of life during the study period.

Analysis of the quality of life was assessed with the SF-36 questionnaire at baseline, treatment week 12 and 24 (Group A only) as well as at follow-up 12 and 24. At all time points we observed no statistical significant differences between Group A and B. As expected under therapy with PEG-IFN and ribavirin in both groups the mean physical as well as mental health score was lower during therapy compared to the phase of follow-up. However, in both groups the patients indicated their quality of life inferior to the reference population with a mean of 50 even in the phase of follow-up. During the first 12 weeks of follow-up the quality of life improved in both groups. Importantly, during week 12 and 24 of follow-up no further improvement was observed (Fig 6 and Table 7).

**Table 7:** SF-36 Physical and Mental Health.

	SF-36 Physical Health		p-value	SF-36 Mental Health		p-value
	Group A (24 weeks) n = 50	Group B (12 weeks) n = 49		Group A (24 weeks) n = 50	Group B (12 weeks) n = 49	
**Baseline**			0.4061			0.9634
MedianRangeInterquartile rangen =	39.8822.10–57.3515.0637	39.7924.20–60.2111.5932		35.9820.40–60.3223.0837	36.9520.65–57.4419.0932	
**W12**			0.2347			0.5369
MedianRangeInterquartile rangen =	38.1819.14–55.7815.0930	43.5028.86–57.0116.8632		36.7819.15–61.6723.2930	35.6116.62–57.7718.4832	
**W24**						
MedianRangeInterquartile rangen =	40.3017.54–58.8519.3236			39.2420.91–57.8325.4236		
**FU12**			0.1883			0.5734
MedianRangeInterquartile rangen =	49.7725.19–58.9913.2831	53.6426.69–61.0110.8227		51.7618.81–60.3015.5319	49.0117.68–59.9318.8327	
**FU24**			0.2755			0.8994
MedianRangeInterquartile rangen =	52.5230.71–59.2613.6730	54.5432.06–60.799.2024		50.0017.72–60.1421.8230	50.7917.40–57.819.0724	

## Discussion

The prospective OPTEX trial investigated the efficacy of treatment prolongation to 36–48 weeks with pegylated interferon alpha-2b and ribavirin in patients with genotype 2/3 and non-RVR showed no major improvement of SVR compared to historical data with 24 weeks treatment. However, our study has some limitations. One of these limitations is that the anticipated number of patients for the study was not reached. Initially, the study was powered for 150 patients in total with 75 in each Group. All patients with non-RVR within a nationwide non-interventional registry should be screened for the OPTEX trial. Initially it was planned to enrol 700 patients between June 2008 and December 2010 in the German G2/3 registry. However, despite an extension of the enrolment in the registry of two years and enrolment of more than 1000 patients in the registry, the calculated number of patients was not achieved. Based on these results roughly 1500 patients had to be enrolled in the registry in order to achieve the enrolment of 150 patients with non-RVR for this trial. Major reasons for the low number of screened patients were the high rate of patients who had not been treated for 24 weeks, the high number of patients without week 4 response data and the low frequency of non-RVR in HCV genotype 2 and 3 patients (Fig 3). This limitation can be seen as an important results indicating that dual therapy of PEG-IFN/RBV is highly effective in the majority of G2/3 patients [15]. Another limitation is, that IL28B (IFNL3) testing was not part of the study protocol as the study was initiated before the discovery that IL28B is predictive for IFN response in chronic hepatitis C [21]. However, we could retrospectively test IL28B in about 2/3 of the patients. The SVR rate was not lower in the patients with the unfavourable IL28B-rs8099960-*T genotypes. The number of patients is too low to draw any conclusion on IL28B genotypes but we think that in patients with non-RVR we have already pre-selected the difficult to treat patients. Thus, the IL28B genotype may not have this great impact as in other cohorts.

Overall only 119 patients were eligible for the screening but again not all (n = 104) individuals were screened mainly due to the fear of further side effects for additional 12 or 24 weeks caused by study medication. This, on the other side, emphasizes the need for less toxic IFN free therapies. The analysis of the SF-36 questionnaire clearly showed the impaired quality of life in all patients within this study. Indeed, many physicians decided to wait for sofosbuvir regimens in G2/3 patients. During the study period, new data for sofosbuvir have been released and showed promising results in several clinical trials in all HCV genotypes even in genotype 3 [7]. Treatment of chronic hepatitis C has dramatically changed with the development of DAA. For genotype 2/3 the NS5B polymerase inhibitor sofobuvir is now approved and effective. However, many countries around the world have limited access to sofosbuvir in 2014. Also for countries with access to DAA, the treatment for 12–24 weeks is expensive with 60,000 to 120,000 € per treatment. The old standard treatment PEG-IFN/RBV can also be highly effective in patients with Genotypes 2/3, especially in patients with rapid virological response [15]. On the other hand, patients without RVR showed lower SVR rates after 24 weeks of treatment and treatment prolongation as shown here does not substantially increase the success rate. Only patients that are fully adherent and complete 48-week treatment with PEG-IFN/RBV may benefit from this prolonged treatment approach. Interestingly, females treated for 48 weeks were the only subgroup that reached nearly 90% SVR in the ITT analysis, which is nowadays the SVR rate to aim for (Table 5). However, the tolerability is difficult to predict early during treatment and based on the current development in the HCV field, we would like to propose a response guided treatment algorithm for non-cirrhotic naïve G2/3 patients without contraindications for PEG-IFN. This approach includes treatment with PEG-IFN/RBV of easy to treat G2/3 patients and adding or switch to sofosbuvir in the non-RVR patients. IL28B genotyping may be helpful in selecting patients which are more likely to achieve RVR and thus being the adequate patient for this approach [22,23]. If dual therapy is initiated, this therapy should be stopped if HCV RNA is positive after 4 weeks (independent of IL28B genotype) because even longer treatment does not increase overall SVR sufficiently. In that case, sofosbuvir based DAA treatment should be started. This response-guided approach may be especially important for patients with genotype 3 because the DAAs are least effective for genotype 3 and our study enrolled mainly patients with genotype 3.

In conclusion, approximately ¼ of G2/3 patients treated with dual PEG-IFN/RBV did not achieve RVR in a real world setting. Treatment duration longer than 24 weeks did not result in higher SVR compared to a historical control group and should therefore not be considered in the era of DAA.

## Supporting Information

S1 Text (PDF)

S2 Text (PDF)

S3 Text (PDF)
