# Supplementary material for: Treatment Extension of Pegylated Interferon Alpha and Ribavirin Does Not Improve SVR in Patients with Genotypes 2/3 without Rapid Virological Response (OPTEX Trial): A Prospective, Randomized, Two-Arm, Multicentre Phase IV Clinical Trial
Source: PLoS One. 2015 Jun 9;10(6):e0128069. doi: 10.1371/journal.pone.0128069 (PMC4461366; doi:10.1371/journal.pone.0128069)
Supplement: S1 Text — (PDF) [file pone.0128069.s001.pdf]

MHH
Medizinische Hochschule Hannover

Ethikkommission
Vorsitzender:
Prof. Dr. H. D. Tröger

Sekretariat:
Rita Landowski
Telefon: 0511 532-3443
Liane Höft
Telefon: 0511 532-9812
Fax: 0511 532-5423
landowski.rita@mh-hannover.de

Carl-Neuberg-Straße 1
30625 Hannover
Telefon: 0511 532-0
www.mh-hannover.de

MHH Ethikkommission OE 9515
30623 Hannover

HCTC Hannover Clinical Trial Center GmbH
Frau Celine Rasche-Schürmann
Carl-Neuberg-Straße 1
30625 Hannover

EINGANG
02. Okt. 2008
HCTC GmbH

30.09.08/La

Nr. 5012M
EudraCT-Nr. 2008-000706-36
OPtimization of treatment for patients with chronic hepatitis C infected with HCV-genotype 2 or 3: 12 vs. 24 weeks of Treatment EXtension for patients without rapid virological response (OPTEX 2/3)

Sehr geehrte Frau Rasche-Schürmann,

die Ethikkommission der Medizinischen Hochschule Hannover hat die Federführung für die o. g. Multicenterstudie und demzufolge am 22.08.08 und nach Einreichung der überarbeiteten Unterlagen über diese Studie abschließend beraten. Die Beratung erfolgte im Benehmen mit den unten aufgeführten lokalen Ethikkommissionen. Dabei wurden die im Anhang 1 erwähnten Unterlagen berücksichtigt. Die Ethikkommission erteilt eine zustimmende Bewertung, da Versagensgründe nach der 12. AMG-Novelle nicht vorlagen. Die klinische Prüfung ist ärztlich vertretbar, ein Nutzen für die Heilkunde ableitbar.

Die zustimmende Bewertung gilt für die in Anhang 2 aufgeführten Prüfzentren. Die in Anhang 2 aufgeführten lokalen Ethikkommissionen haben der Durchführung in den jeweiligen Prüfzentren zugestimmt.

Die Ethikkommission weist darauf hin, dass die ärztliche und juristische Verantwortung bei den jeweiligen Prüfärzten verbleibt.

An der abschließenden Beratung und Beschlussfassung haben die in Anhang 3 aufgeführten Mitglieder der hiesigen Ethikkommission teilgenommen. Es wird bestätigt, dass Prüfärzte, die an der o. g. Studie beteiligt sind, nicht an der Abstimmung teilgenommen haben.

Die Ethikkommission gibt folgende allgemeine Hinweise:

1. Die Zustimmende Bewertung ist für die in Anhang 2 aufgeführten Personen/ Einrichtungen gültig. Eine Änderung der lokalen Prüfstellen ist der hiesigen Ethikkommission und der jeweils lokal zuständigen Ethikkommission mitzuteilen.

2. Auf die Einhaltung einschlägiger Gesetze und Rechtsvorschriften wird hingewiesen. Die nach Rechtslage notwendigen Unterrichtungen (u. a. Prüfplanänderungen, entsprechende Zwischenfallsereignisse, neue Datenlage, Nachmeldung von Prüfzentren, Jahresbericht, Abschlussbericht) sind den jeweils zuständigen Ethikkommissionen unverzüglich vorzulegen.
3. Die Ethikkommission bestätigt, dass sie auf Grundlage nationaler Gesetze, Vorschriften sowie der GCP/ICH-Richtlinie arbeitet.
4. Eine Kopie dieser Stellungnahme wird der zuständigen Behörde zugeleitet.
5. Gegen die vorliegende Stellungnahme kann innerhalb von einem Monat nach Bekanntmachung Widerspruch erhoben werden. Der Widerspruch ist schriftlich bei der Geschäftsstelle der federführenden Ethikkommission zusammen mit einer Begründung einzureichen.

Mit den besten Grüßen bin ich
Ihr

Prof. Dr. H. D. Tröger
Vorsitzender der Ethikkommission
(nach Diktat verreist)

i. V. Tanja Germerott
Geschäftsf. Ärztin

Nachrichtlich:
Zweitvotierende Ethikkommissionen

BfArM

Anhang 1

Folgende Unterlagen wurden vorgelegt:
Modul 1 + 2, Checkliste
Bestätigungsschreiben Eudract-Nummer
Versicherungsnachweis
Vertragsmodalitäten
Qualifikationsnachweis der Prüfstellen, CV, Financial Disclosure Form der Prüfärzte
Fachinformationen
Patienteninformation Version 02 und Einwilligungserklärung Version 02, 03 und 04
Studienprotokoll Version 21.04.08
Liste der Beteiligten Prüfzentren und Ethikkommissionen

Anhang 2

Das Votum ist gültig für die nachfolgend aufgeführten Prüfzentren:

- Prof. Dr. M. Manns (LKP),Dr. M. Cornberg, Abt. für Gastroenterologie, Hepatologie und Endokrinologie, Hannover
- PD Dr. H. Wasmuth, Medizinische Klinik III, Universitätsklinikum Aachen
- PD Dr. H. Hinrichsen, Gastroenterologische Gemeinschaftspraxis, Kiel
- Prof. Dr. T. Berg, Med. Klinik Gastroenterologie/Hepatologie, Charité Campus Virchow-Klinikum
- Dr. J Gölz, Berlin
- Dr. C. John, Ärztehaus Leipziger Straße, Berlin
- Dr. U. Meyer, Berlin
- Dr. B. Möller, Hepatologische Schwerpunktpraxis im bng., Berlin
- Prof. Dr. U. Spengler, Medizinische Klinik und Polklinik I, Bonn
- PD Dr. J. Ockenga, Klinikum Bremen-Mitte gGmbH, Innere Med. II, Bremen
- Dr. S. Wollschläger, Krankenhaus Dresden-Friedrichstadt III. Med.Abt., Dresden
- Dr. H. Carls, F. Huber, Fachärztl Gemeinschaftspraxis, Düsseldorf
- Dr. W. Norgauer, St.-Theresien-Krankenhaus, Innere Medizin, Nürnberg
- Prof. Dr. G. Gerken, Dr. S. Haag, PD Dr. A. Canbay, Prof. Dr. J. Schlaak, Dr. Ch. Jochum, Universitätsklinikum Essen, Zentrum f. innere Medizin, Essen
- Dr. M. Leuschner (HP) Dr. H.-J. Cordes, Hepatologische Schwerpunktpraxis im bng, Offenbach
- Prof. Dr. S. Zeuzem, Dr. Katrin Wunder, Dr. Nicole Forestier, Klinikum der J.W. Goethe-Universität, Med. Klinik I, Frankfurt/Main
- Prof. Dr. M. Rössle, Praxis Zentrum Gastroenterologie und Endokrinologie, Freiburg
- Dr. A. Stoehr, Prof. Dr. A. Plettenberg, Christine Czaja-Harder, St. Unger, Dr. M. Sabranski, Dr. Karen Olah, ifi – Studien und Projekte GdR an der Asklepios Klinik St. Georg, Hamburg
- Prof. Dr. A. Lohse, Dr. St. Lüth, Universitätsklinikum Hamburg-Eppendorf, Klinik für Innere Medizin, Hamburg
- Prof. Dr. J. Arnold, Diakoniekrankenhaus, Med. Klinik II, Rotenburg/Wümme
- Dr. S. Holm, Hannover
- Dr. C. Eisenbach, Medizinische Fakultät der Universität Heidelberg, Innere Medizin IV, Heidelberg
- Prof. Dr. A. Stallmach, Dr. A. Hermann, Klinik für Innere Medizin der FSU, Innere Med. II, Jena
- Dr. R. Günther, UK S.H, Campus Kiel, Klinik für Allgemeine Innere Medizin,
- Dr. J. Wiegand, Universitätsklinikum Leipzig, Medizinische Klinik und Poliklinik II, Leipzig
- Dr. T. Witthöft, Universitätsklinikum Schleswig-Holstein, Campus Lübeck, Med. Klinik I, Abt. Gastroenterologie, Lübeck
- Prof. Dr. P. Malfertheiner, Otto-von-Guericke Universität Magdeburg, Magdeburg
- Prof. Dr. M. Singer, Univ.-Klinikum Mannheim, Med. Klinik, Mannheim
- Prof. R. Zachoval, Klinikum Großhadern, Medizinische Klinik 2, München
- Dr. H. Busch, Centrum für Interdisziplinäre Medizin, Münster
- Dr. D. Hüppe, Prof. Dr. H. Hartmann, Dr. Giesela Felten, Hepatologische Schwerpunktpraxis im bng, Ärzthaus am Evangelischen Krankenhaus, Herne

- PD Dr. A. Lügering, Universitätsklinikum Münster, Medizinische Klinik und Poliklinik B, Münster
- Dr. C. Roggel, Hepatologische Schwerpunktpraxis im bng, Minden
- Dr. E. Zehnter, Hepatologische Schwerpunktpraxis im bng, Dortmund
- Dr. R. Wiest, PD Dr. Cornelia Gelbmann, Uniklinikum Regensburg, Klinik und Poliklinik für Innere Med. I, Regensburg,
- Prof. Dr. F. Lammert, Dr. F. Grünhage, N. Afan, Universitätskliniken des Saarlandes, Innere Medizin II / Gastroenterologie, Homburg/Saar
- Dr .R. Link, St. Josefs-Klinik, Medizinische Klinik, Offenburg
- Dr. A. Trein, Stuttgart
- Dr. S. Kaiser, Universitätsklinikum Tübingen, Med. Klinik I, Tübingen
- Dr. N. Dikopoulos, Dr. D. Klass, Universitätsklinikum Ulm, Abt. Für Innere Medizin I, Ulm
- Prof. Dr. H. Klinker, Med. Poliklinik der Universität Würzburg, Standort Luitpoldkrankenhaus, Bau 18, Würzburg
- Prof. Dr. M. Kraus, Kreiskliniken Burghausen/Altötting, Med. Klinik II, Burghausen/Altötting
- Prof. Dr. M. Gregor, Dr. P. S. Kaiser, Universitätsklinikum Tübingen

Folgende lokale Ethikkommissionen haben sich zustimmend an der Beratung beteiligt:

Ethikkommission der Medizinischen
Fakultät des Universitätsklinikums
der RWTH Aachen
Pauwelsstraße 30
52074 Aachen

Ethikkommission der
Ärztekammer Schleswig-Holstein
Bismarckallee 8-12
23795 Bad Segeberg

Ethikkommission des Landes Berlin beim
Landesamt für Gesundheit u. Soziales
Sächsische Str. 28
10707 Berlin

Ethikkommission an der Medizinischen
Fakultät der Rheinischen
Friedrich-Whilhelms-Universität Bonn
Reuterstraße 2b
53113 Bonn

Ethikkommission des Landes Bremen
Klinikum Bremen-Mitte gGmbH
Sankt-Jürgen-Straße 1d
28177 Bremen

Ethikkommission der
Sächsischen Landesärztekammern
Schützenhöhe 16-18
Postfach 100465
01099 Dresden

Ethikkommission der
Ärztekammer Nordrhein
Tersteegenstraße 9
40474 Düsseldorf

Ethikkommission der
Bayerischen Landesärztekammer
Mühlbaurstraße 16
81677 München

Ethikkommission der Med. Fakultät der
Universität Duisburg-Essen
Universitätsklinikum Essen
Robert-Koch-Straße 9-11
45147 Essen

Ethikkommission der
Landesärztekammer Hessen
Im Vogelsgesang 3
60488 Frankfurt/Main

Ethikkommission der
J. W.-Goethe-Universität Frankfurt
Theodor-Stern-Kai 7
60590 Frankfurt am Main

Ethik-Kommission der
Albert-Ludwigs-Universität Freiburg
Elsässer Str. 2m, Haus 1 A
79110 Freiburg

Ethikkommission der
Ärztekammer Hamburg
Humboldtstraße 67a
22083 Hamburg

Ethikkommission der
Landesärztekammer Niedersachsen
Berliner Allee 20
30175 Hannover

Ethikkommission der Medizinischen
Fakultät Universität Heidelberg
Alte Glockengießerei 11/1
69115 Heidelberg

Ethikkommission der
Friedrich-Schiller-Universität Jena
Bachstraße 18
07740 Jena

Ethikkommission der Medizinischen
Fakultät der CAU zu Kiel
Schwanenweg 20
24105 Kiel

Ethikkommission an der Med. Fakultät
der Universität Leipzig
Härtelstraße 16-18
04107 Leipzig

Ethikkommission der Med. Fakultät der
Universität zu Lübeck
Ratzeburger Allee 160
23538 Lübeck

Ethikkommission der Med. Fakultät der
Otto-von-Guericke-Universität Magdeburg
Leipziger Straße 44
39120 Magdeburg

Medizinische Ethikkommission II der
Fakultät für klinische Medizin
Ruprecht-Karls-Universität Heidelberg
Maybachstraße 14-16
68169 Mannheim

Ethikkommission der
Medizinischen Fakultät der
Ludwig-Maximilians-Universität
Marchioninistraße 15
81377 München

Ethikkommission der
Ärztekammer Westfalen-Lippe
und der Med. Fakultät der WWU Münster
Von-Esmarch-Straße 62
48149 Münster

Ethikkommission an der
Universität Regensburg
Franz-Josef-Strauß-Allee 11
93053 Regensburg

Ethikkommission der
Ärztekammer des Saarlandes
Postfach 10 02 62
66002 Saarbrücken

Ethikkommission der
Landesärztekammer Baden-Württemberg
Postfach 700 361
70573 Stuttgart

Ethikkommission der
Universität Tübingen
Schleichstraße 8
72076 Tübingen

Ethikkommission der Universität Ulm
Helmholtzstraße 20 (oberer Eselsberg)
89081 Ulm

Ethik-Kommission der Med. Fakultät
der Universität Würzburg
Josef-Schneider-Straße 2
97080 Würzburg

Anhang 3

Folgende Mitglieder haben an der Beratung des o.g. Antrages mitgewirkt:

Prof. Dr. Alfred Berger, em. Leiter der PHW Oststadt-Krankenhaus
Prof. Dr. Anibh M. Das, Päd. Nierenerkrankungen, Kinderklinik der MHH
Prof. Dr. Dr. h.c. E. Deutsch, Abt. für Arzt- und Arzneimittelrecht, Universität Göttingen
Dr. Stefan Engeli, Klinische Pharmakologie der MHH
Prof. Dr. J. Frölich, em. Leiter der Abt. Klin. Pharmakologie der MHH
Dr. J. Graubner, Arzt f. Allgemeinmedizin
Prof. Dr. H. Hecker, Komm. Leiter der Abt. Biometrie der MHH
Prof. Dr. J. Jordan, Abt. Klin. Pharmakologie der MHH
Frau Prof. Dr. B. Lohff, Leiterin der Abt. Geschichte, Ethik und Philosophie der Medizin der MHH
Prof. Dr. H. D. Tröger (Vorsitzender), Leiter der Abt. Rechtsmedizin der MHH
