# Supplementary material for: Treatment Extension of Pegylated Interferon Alpha and Ribavirin Does Not Improve SVR in Patients with Genotypes 2/3 without Rapid Virological Response (OPTEX Trial): A Prospective, Randomized, Two-Arm, Multicentre Phase IV Clinical Trial
Source: PLoS One. 2015 Jun 9;10(6):e0128069. doi: 10.1371/journal.pone.0128069 (PMC4461366; doi:10.1371/journal.pone.0128069)
Supplement: S2 Text — (PDF) [file pone.0128069.s002.pdf]

KOMPETENZNETZ HEPATITIS
CLINICAL STUDY PROTOCOL

-Confidential-

STUDY TITLE:

OPtimization of treatment for patients with chronic hepatitis C infected with HCV-genotype 2 or 3: 12 vs. 24 weeks of Treatment EXtension for patients without rapid virological response (OPTEX 2/3)

VERSION 03, 30.11.2011, including Amendment 06

Protocol Number: P05498
EudraCT-Nummer 2008-000706-36

SPONSOR:
Competence Network Viral Hepatitis (Hep-Net)
c/o Department of Gastroenterology, Hepatology, and Endocrinology
Prof. M. P. Manns, M.D.
Hannover Medical School
Carl-Neuberg Strasse 1
30625 Hannover
Germany
Tel.: +49 (0)511-532 3305, Fax: +49 (0)511-532 6820
Email: manns.michael@mh-hannover.de

STUDY COORDINATORS:
Hep-Net Study House, Hannover Medical School:
Prof. Dr. med. M.P. Manns (LKP)
Dr. med. M. Cornberg
PD Dr. med. H. Wedemeyer
Benjamin Heidrich, M.D.
Tel.:+49(0)511-532 6817, Fax:+49(0)511-532 6820,
Email: cornberg.markus@mh-hannover.de

GLOSSARY OF ABBREVIATIONS

AE	adverse event
ALT (GPT)	alanine aminotransferase
AST (GOT)	aspartate aminotransferase
AMG	Arzneimittelgesetz
BP	blood pressure
CRF	case report form(s)
EMEA	European Medicines Agency
ECG	electrocardiogram
EVR	early virological response
FDA	Food and Drug Administration
FU	Follow-up visit
GCP	Good Clinical Practice
HAV	hepatitis A virus
HBV	hepatitis B virus
HCV	hepatitis C virus
HIV	human immunodeficiency virus
ICH-GCP	International Conference of Harmonisation - Good Clinical Practice
IEC	Independent Ethics Committee
IFN	interferon alpha
INR	international normalised ratio
ITT	intention to treat
IU	international units
IUD	intrauterine device
LKP	Leiter der klinischen Prüfung (Coordinating Investigator)
MEDRA	Medical Dictionary for Regulatory Activities Terminology
NYHA	New York Heart Association
PEG	polyethylene glycol
QW	once weekly
RBC	red blood cell
RBV	ribavirin
RNA	ribonucleic acid
RVR	rapid virological response
SAE	serious adverse event
SC	subcutaneous
SmPC	Summary of Product Characteristics
SVR	sustained virological response
TSH	thyroid stimulating hormone
TW	treatment week
ULN	upper limits of normal
WBC	white blood cell
WK	week

1 BACKGROUND AND RATIONALE ... 2
1.1 Chronic Hepatitis C ... 2
1.2 Study drugs – Current standard treatment ... 2
1.3 Rationale for the Study ... 3
2 OBJECTIVES ... 3
3 STUDY DESIGN ... 3
3.1 Overview of Study Design ... 3
3.2 Endpoints ... 4
3.3 Assignment to Treatment Groups ... 5
4 STUDY POPULATION ... 5
4.1 Target Population ... 5
4.2 Inclusion criteria ... 5
4.3 Exclusion criteria ... 5
5 SCHEDULE OF ASSESSMENTS AND PROCEDURES ... 6
5.1 Study Procedures / Assessments ... 7
5.2 Screening Examination and Eligibility Screening Form ... 7
6 DRUG INFORMATION AND DOSE MODIFICATIONS ... 8
6.1 Drug information ... 8
6.2 Dosage of investigational drugs ... 8
7 SAFETY ISSUES ... 9
7.1 Warnings and Precautions ... 9
7.2 Adverse Events ... 9
7.3 Serious Adverse Events ... 9
7.4 Reporting of SAE's ... 10
7.5 Management of adverse events ... 10
7.6 Benefit-Risk-Assessment ... 12
8 STATISTICAL METHODS AND SAMPLE SIZE ... 13
8.1 Statistical Methods ... 13
8.2 Sample Size Justification ... 14
8.3 Historical control group ... 14
9 DATA QUALITY ASSURANCE ... 15
10 ETHICAL ASPECTS ... 15
10.1 Local Regulations/Declaration of Helsinki ... 15
10.2 Patient Information and Informed Consent ... 15
10.3 Insurance ... 16
10.4 Independent Ethics Committees (IEC) ... 16
10.5 Financing ... 16
11 CONDITIONS FOR MODIFYING THE PROTOCOL ... 16
12 SUBJECT COMPLETION ... 16
12.1 Procedures for handling patients lost to follow-up and drop-outs ... 16
12.2 Reasons for drop-out ... 17
13 STUDY DOCUMENTATION, electronic CRF AND RECORD KEEPING ... 17
13.1 Investigator's Files/Retention of Documents and Source Documents ... 17
13.2 Audits and Inspections ... 18
13.3 Case Report Forms (CRFs) ... 18
14 MONITORING THE STUDY ... 18
14.1 Direct Source Data Verification ... 18
15 CONFIDENTIALITY OF TRIAL DOCUMENTS AND PATIENTS RECORDS ... 18
16 PUBLICATION OF DATA ... 18
17 SIGNATURES ... 19
18 REFERENCE LIST ... 20
19. STUDY SET-UP ... 22

1 BACKGROUND AND RATIONALE

1.1 Chronic Hepatitis C

More than 120 million people world-wide are chronically infected with the hepatitis C virus (HCV) [18], a RNA virus belonging to the family flaviviridae which was discovered in 1989 [2]. HCV is transmitted primarily through exposure to blood products and intravenous drug use. 50-90% of patients with acute HCV infection develop a persistent infection. Heterogeneity is high, there are 6 HCV-genotypes and more than 90 subtypes. The chronic sequelae of chronic HCV infection are liver fibrosis, cirrhosis, and hepatocellular carcinoma [11,13]. In addition to liver disease, HCV infection has been associated with a wide variety of extrahepatic manifestations such as mixed cryoglobulinaemia, membranoproliferative glomerulonephritis and porphyria cutanea tarda [10,16].

Measures to prevent HCV infection such as blood screening programs led to a decline in HCV incidence in the developed world. However, despite prevention measures, we still anticipate an increasing number of patients with sequelae of HCV infection in the following 10-20 years [9,11].

During the last 15 years there has been an enormous achievement in the diagnosis, management, and therapy of hepatitis C. Analysis of HCV-genotypes, quantification of HCV-RNA viral load, and calculation of viral kinetics allow better management of patients with chronic hepatitis C. Current standard treatment of HCV with pegylated interferon alpha (PEG-IFN) and ribavirin has been optimized and the first direct antiviral drugs are in development and may lead to chance to cure chronic hepatitis C in the majority of cases [3,17].

1.2 Study drugs – Current standard treatment

Pegylated Interferon alpha-2b (PegIntron®)

Before the identification of the Hepatitis C Virus (HCV) as the infectious agent for non-A, non-B hepatitis it turned out that Interferon alpha (IFN) may lead to normalization of transaminases and improvement of liver histology [12]. Due to the identification of HCV it became possible to measure the success of therapy as a long lasting disappearance of HCV-RNA from serum, a so-called sustained virological response (SVR). Since that time the SVR rate has increased from 5-20% with an IFN monotherapy to 40%-50% with the combination of IFN and ribavirin (11;12). The development of pegylated interferon alpha (PEG-IFN) added a new milestone to the treatment of chronic hepatitis C. Pegylation of the IFN allows a once weekly administration due to an improved pharmacokinetic profile. PEG-IFN/ribavirin combination therapy improved the overall SVR to 54-63% [4]

PEG-IFN alpha-2b is given as a subcutaneous injection adjusted for body weight (1.5 µg/kg once weekly). Side effects of PEG-IFN alpha-2b include fatigue, flu-like symptoms, neuropsychiatric symptoms, autoimmune phenomena, and haematological abnormalities which may lead to dose modifications or therapy discontinuation. PegIntron® was approved by the EMEA in 2002 for the treatment of chronic hepatitis C.

Ribavirin (Rebetol®)

Ribavirin should be administered according to the bodyweight of the patient. A retrospective analysis of the large PEG-IFN alpha-2b/ribavirin pivotal trial revealed that the optimal ribavirin dose is at least 10.6 mg/kg [15]. Therefore ribavirin (Rebetol®) is recommended at a concentration of approximately 11 mg/kg body weight in combination with PEG-IFN alpha-2b (see dosing). The main side effect of ribavirin is a dose dependent haemolysis which may require dose modification. Rebetol® was approved by the EMEA in 1999 for the treatment of chronic hepatitis C.

The current standard treatment duration is dependent on the HCV-genotype. Patients with the HCV-genotype 1 are treated for 48 weeks and patients with the HCV-genotypes 2 and 3 are treated only for 24 weeks [17].

1.3 Rationale for the Study

The main discussion within the scientific community is to optimize the current standard treatment of chronic hepatitis C (CHC) to improve the response rates, in particular the treatment duration. There are two different concepts to optimize the treatment duration. While some patients may be treated for a shorter period of time to reduce costs and side-effects, others may need longer treatment to improve the response rates.

Many studies have been investigating reductions in treatment duration for HCV-genotypes 2 and 3 to 16, 14, or even 12 weeks. The first reported results are promising, but it turns out that individual factors need to be considered when treating patients for less than 24 weeks. The rapid virological response (RVR) after 4 weeks of therapy (HCV-RNA negative in the serum at treatment week (TW) 4) is one of the critical factors that are associated with the success of a shorter therapy. Only patients who showed RVR at week 4 had high SVR rates after 16 weeks [20], 14 weeks [5], or even after 12 weeks of therapy [14], whereas those without RVR had lower response rates, even with the 24-week schedule. In addition to the RVR other factors are associated with the response in patients with HCV-genotypes 2 and 3. These are the baseline viral load [5,19,20] and the presence of liver cirrhosis [1]. In conclusion, patients with HCV-genotype 2 and 3 and low viral load who have a RVR after 4 weeks of therapy can be treated for less than 24 weeks and patients without RVR (especially HCV-genotype 3 and high viral load) may be treated even for more than 24 weeks. However, the optimal treatment duration for patients without RVR is not known until now.

In this study we intend to treat patients with chronic hepatitis C of genotype 2 or 3 having characteristics associated with poor treatment response for additional 12 or 24 weeks beyond the standard treatment of PEG-IFN alpha-2b plus ribavirin.

2 OBJECTIVES

The objective of this study is to compare the efficacy of a treatment extension of 12 versus 24 weeks in patients with HCV-genotypes 2 and 3 who are treated with 1.5 µg/kg PEG-IFN alpha-2b and 800-1400 mg ribavirin (standard dose) for 24 weeks (standard duration) and who are not HCV-RNA negative (< 15 IU/ml) after 4 weeks of standard treatment

3 STUDY DESIGN

3.1 Overview of Study Design

This is a German open label multi center randomized phase IV trial to assess the efficacy of 12 versus 24 weeks of extended treatment in HCV-G2/3 patients with an ongoing standard treatment with PEG-IFN alpha-2b and ribavirin.

150 patients shall be enrolled. Estimated study start will be July 1st 2008; period for recruiting is planned to be 18 months. The recruitment period will be extended until December 31st 2012 and last follow up visit will be performed in June 2013.

Approximately 30 centers specialised in the treatment of hepatitis C will take part in the study. The number of patients per center is planned to be at least 3.

Patients with HCV-genotype 2 and 3 are eligible if they are non rapid virological responders (HCV-RNA positive after 4 weeks of treatment).

Group A: PegIntron® 1.5 µg/kg once weekly (QW) subcutaneous (sc) plus Rebetol® 800-1400 mg per os divided in 2 daily doses for additional 24 weeks beyond standard treatment with 24 weeks follow-up

Group B: PegIntron® 1.5 µg/kg QW sc plus Rebetol® 800-1400 mg per os divided in 2 daily doses for additional 12 weeks beyond standard treatment with 24 weeks follow-up

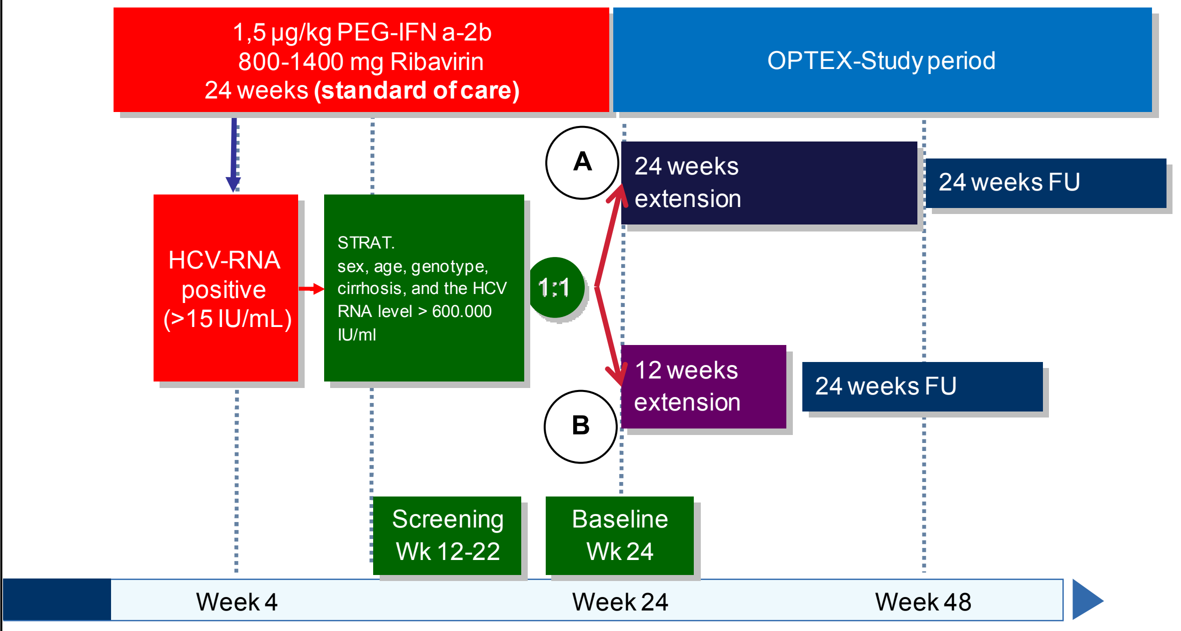

Figure 3: Study Design OPTEX 2/3

3.2 Endpoints

Primary endpoint is

- Reduction of Relapse rate (HCV-RNA positive in serum by a standard HCV-PCR with a detection limit of at least 15 IU/ml) 24 weeks after the end of treatment und thus improvement of sustained virological response rates (SVR)

Secondary endpoints are

- Virological response rates (HCV-RNA negative in serum by a standard HCV-PCR with a detection limit of at least 15 IU/ml) at the end of therapy
- Biochemical responses as determined by ALT and AST levels at the end of treatment and at the end of follow up.
- Severity and frequency of adverse events (AE)
- Analysis of quality of life (with questionnaire SF-36)

3.3 Assignment to Treatment Groups

150 patients with chronic hepatitis C virus infection of the genotypes 2 and 3 shall be enrolled. Patients will be randomized 1:1 in group A (75 patients) and B (75 patients). Stratification factors will be sex, age, genotype, cirrhosis, and the HCV RNA level > 600.000 IU/ml before the ongoing therapy.

4 STUDY POPULATION

4.1 Target Population

The target population is male and female with HCV-genotype 2 or 3 chronic hepatitis c virus infection, ≥ 18 years of age.

4.2 Inclusion criteria

For inclusion into the study, a patient must satisfy the following criteria:

1. Male or female patients with HCV-genotype 2/3 chronic hepatitis C documented by detectable plasma HCV RNA (> 15 IU/mL) and positivity of anti-HCV antibodies
2. Age ≥ 18 years
3. Compensated liver disease (Child-Pugh Grade A clinical classification)
4. Negative urine or blood pregnancy test (one of the both; for women of childbearing potential) documented within the 24-hour period prior to the first dose of study drug. Additionally, all fertile males and females must be using two forms of effective contraception during treatment and during the 7 months after treatment end. This includes using birth control pills (no interaction with investigational drugs), IUDs, condoms, diaphragms, or implants, being surgically sterilized, or being in a post-menopausal state. At least one contraception method must be of barrier method
5. Ongoing treatment with 1.5 µg/kg Peg-Interferon alpha-2b (PegIntron®) and > 10.6 mg/kg ribavirin (Rebetol®)
6. No rapid virological response (HCV-RNA positive after week 4 of the ongoing therapy)
7. Willingness to give written informed consent and willingness to participate to and to comply with the study protocol

4.3 Exclusion criteria

Patients with any of the following criteria will be excluded from the study:

1. Women with ongoing pregnancy or breast feeding
2. Male partners of women who are pregnant
3. Coinfection with HIV or HBV
4. History or other evidence of a medical condition associated with chronic liver disease other than HCV associated (e.g., hemochromatosis, autoimmune hepatitis, alcoholic liver disease, toxin exposures)
5. History or other evidence of bleeding from esophageal varices or other conditions consistent with decompensated liver disease
6. Patients with liver cirrhosis with a lesion suspicious for hepatic malignancy on the screening
7. Absolute neutrophil count (ANC) <750 cells/mm3 at screening
8. Platelet count <50,000 cells/mm3 at screening
9. Hb <10 g/dl at screening

10. Dose modification of Peg-Interferon alpha-2b (PegIntron®) or ribavirin (Rebetol®) during the first 4 weeks of the ongoing therapy
11. Interferon alpha or ribavirin therapy at any time point before the actual ongoing treatment
12. Less than 80% adherence to treatment of the ongoing treatment until randomization (week 20-22 of ongoing treatment)
13. Serum creatinine level >1.5 times the upper limit of normal at screening
14. History of severe psychiatric disease, especially depression (ICD 10 codes F30–F33). Severe psychiatric disease is defined as treatment with an antidepressant medication or a major tranquilizer at therapeutic doses for major depression or psychosis, respectively, for at least 3 months at any previous time. Patients are excluded if any history of suicidal attempts is evident. If hospitalization for psychiatric disease, or a period of disability due to a psychiatric disease are documented, psychiatric consultation is mandatory. Patients with a mild or moderate psychiatric disease (ICD 10 codes F32.0, F32.1, F33.0, F33.1) are only allowed to be included into the trial if a regular monitoring by a psychiatrist is performed during the trial
15. History of a severe seizure disorder or current anticonvulsant use
16. History of immunologically mediated disease (e.g., inflammatory bowel disease, idiopathic thrombocytopenic purpura, lupus erythematosus, autoimmune hemolytic anemia, scleroderma, severe psoriasis, rheumatoid arthritis)
17. History or any other evidence of autoimmune diseases
18. History or other evidence of chronic pulmonary disease associated with functional limitation
19. History of significant cardiac disease that could be worsened by acute anemia (e.g. NYHA Functional Class III or IV, myocardial infarction within 6 months prior to treatment with Peg-Interferon/ribavirin therapy, ventricular tachyarrhythmias requiring ongoing treatment, unstable angina)
20. Evidence of thyroid disease that is poorly controlled on prescribed medications
21. Evidence of severe retinopathy (e.g. CMV retinitis, macular degeneration)
22. History of major organ transplantation with an existing functional graft
23. History or other evidence of severe illness, malignancy or any other conditions which would make the patient, in the opinion of the investigator, unsuitable for the study
24. History of any systemic anti-neoplastic or immunomodulatory treatment (including supraphysiologic doses of steroids and radiation) 6 months prior to the first dose of study drug or the expectation that such treatment will be needed at any time during the study
25. Patients with evidence for tuberculosis
26. Drug abuse within 6 months prior to the first dose of study drug and excessive alcohol consumption. Patients on methadone/polamidone/buprenorphine programs are not excluded
27. Any investigational drug and/or participation in another clinical study prior 6 months to the actual ongoing antiviral treatment
28. Limited contractual capability

5 SCHEDULE OF ASSESSMENTS AND PROCEDURES

All laboratory testing will be performed locally at each site. Each investigator is responsible to submit appropriate laboratory certificates and all ranges of normal values to the HEP-NET study coordinator.

Only laboratories can be involved taking part in quality control programs regularly.

HCV-RNA will be analyzed in a central lab at Hannover Medical School, Clinic for Gastroenterology, Hepatology, and Endocrinology, Prof. Manns, Dr. H. Wedemeyer, Carl-Neubergstr.1, 30625 Hannover; Tel: 0511-532-6814).

5.1 Study Procedures / Assessments

Table 2: Schedule of assessments and procedures

**Assessment**	**Screening**	**Study Treatment Week (TW) 0**	**Study Treatment Week (TW) 4,8,12,16*,20*,24* (*only group A)**	**Follow-up (FU) 4,12,24**
Informed consent	X			
Medical & Medication History	X			
Physical Examination	X			
Vital Signs	X	X	X	X
Adverse Events		X	X	X
Weight	X	X	X	X
ECG	X			
HCV-RNA	X	X	Only end of treatment	X
Haematology	X	X	X	X
Chemistry	X	X	X	X
TSH	X		Only TW 12, 24	Only FU 24
Pregnancy Test	X	X	X	X
Questionnaire SF-36		X	Only TW 12, 24	Only FU 12, 24

Vital Signs: Blood pressure, Heart Rate
Haematology: haemoglobin, RBC, WBC, platelets, coagulation (INR or quick)
Chemistry: creatinine, ALT, AST, Bilirubin, gGT, glucose

A total of approximately 15 ml of blood will be needed at each visit.

5.2 Screening Examination and Eligibility Screening Form

A screening examination should be performed between week 12 and week 22 of ongoing treatment before randomization and study entry (= week -12 and week -2 of study).
During the screening, candidates for the study will be fully informed about the nature of the study and possible benefits and risks, and will receive a copy of the patient information and the informed consent form for review. Candidates must read the patient information and sign the consent form if the investigators have answered all questions to the candidate's satisfaction. Further procedures can begin only after the consent form has been signed. One copy of the signed consent form will be retained by the investigator and a second copy will be given to the candidate.

6 DRUG INFORMATION AND DOSE MODIFICATIONS

6.1 Drug information

The investigational drug Peg-Interferon alpha-2b is available as „PegIntron 50/-80/-100/-120/-150 µg powder inclusive solution within an injector pen. In the event that the pen is not used, the sponsor will provide PEG2b in vials (and sterile water for injection) that allow for dosing equivalent to the pen. Ribavirin is available as „Rebetol 200 mg capsules". Both drugs are licensed in the European Union (EU) by MSD Sharp & Dohme GmbH. PegIntron® is an interferon alpha-2b molecule conjugated with a 12kDa polyerthylene glycol moiety. The characteristic of this formulation is a longer half-life and an improved pharmacokinetic profile compared to the interferon alpha-2b molecule. This allows for a once weekly subcutaneous injection.
Rebetol® is given orally on a daily basis in combination with interferon alpha therapy.

PEG-Interferon alfa-2b (PegIntron®) and Ribavirin (Rebetol®) are approved for the treatment of chronic hepatitis C. Detailed information is given by the Summary of Product Characteristics (SmPC; "Fachinformation").

6.2 Dosage of investigational drugs

PEG-Interferon alpha-2b (PegIntron®) will be given as a subcutaneous injection once weekly weight adjusted as follows#:

**Body Weight**	**Dose**	**Pen or Vial Strength (µg)**	**Injection Volume (ml)**
40-43 kg	60 µg	100 µg	0,3
44-50 kg	70 µg	100 µg	0,35
51-56 kg	80 µg	100 µg	0,4
57-63 kg	90 µg	100 µg	0,45
64-68 kg	100 µg	100 µg	0.5
69-75 kg	105 µg	150 µg	0.35
76-85 kg	120 µg	150 µg	0.4
86-95 kg	135 µg	150 µg	0.45
≥ 96 kg	150 µg	150 µg	0,5

the body weight prior to the ongoing standard therapy defines the dosing

Ribavirin (Rebetol®) will be given as capsules weight adjusted daily as follows#:

**Body weight**	**Dose**	**Capsules/ daily intake**
< 64 kg	800 mg	2-0-2

65-75 kg	1000 mg	2-0-3
76-85 kg	1000 mg	2-0-3
>85 kg	1200 mg	3-0-3
>105 kg	1400 mg	3-0-4

the body weight prior to the ongoing standard therapy defines the dosing

7 SAFETY ISSUES

7.1 Warnings and Precautions

No evidence is available at the time of the completion of the study protocol to the Hep-Net Study Group indicating that special warnings or precautions were appropriate, other than those noted in the SmPC.

7.2 Adverse Events

An adverse event (AE) is any untoward medical occurrence or unfavourable and unintended sign in a subject administered a pharmaceutical product/biologic (at any dose), or medical device, whether or not considered related to the use of that product. Additionally, any event that is associated with or observed in conjunction with a product overdose (whether accidental or intentional) or a product abuse and/or withdrawal is also considered an adverse event.

All AEs encountered during the clinical study will be reported on the AE page of the Case Report Form (CRF). Intensity of adverse events will be graded on a four-point scale (mild, moderate, severe, life threatening) and reported in detail as indicated on the CRF.

The investigator must assess the relationship of any adverse event to the use of study drug, based on available information, using the following guidelines (Table 3):

Table 3. Guidelines for Assessing the Relationship of an Adverse Event to Treatment

Unlikely related:	no temporal association, or the cause of the event has been identified, or the drug, biological, or device cannot be implicated
Possibly related:	temporal association, but other etiologies are likely to be the cause; however, involvement of the drug, biological, or device cannot be excluded
Probably related:	temporal association, other etiologies are possible, but unlikely

7.3 Serious Adverse Events

A serious adverse event (SAE) is any event that results in any of the following outcomes:

- is fatal
- is life-threatening (life-threatening is defined as the patient was at immediate risk of death from the AE as it occurred)
- is significantly or permanently disabling
- requires in-patient hospitalization, or prolongs hospitalization
- is a congenital anomaly or birth defect

Important medical events that may not result in death, be life-threatening, or require hospitalization may be considered a serious adverse drug reaction when, based upon appropriate medical judgment, they may jeopardize the patient or subject and may require medical or surgical intervention to prevent one of the outcomes listed in this definition. Examples of such medical events include allergic bronchospasm requiring intensive treatment in an emergency room or at home, blood dyscrasias or convulsions that do not result in inpatient hospitalization, or the development of drug dependency or drug abuse.

7.4 Reporting of SAE's

Any SAE that occurs while treatment or aftercare whether or not deemed drug-related or expected, should be reported within 24 hours after knowledge by the treating physician to his/her local regulatory or drug safety reporting agency (Sponsor Hep-Net) along with a copy to MSD Sharp & Dohme GmbH, Division Pharmacovigilance. Also SAEs, that occur within 30 days after the last application of the study medication or within 30 days after the last medical check-up need to be reported. In case of pregnancy of a female patient in the study or of a female partner of a male patient participating in the study this must be handled in the same way – it must be reported as if it would be an SAE. If the pregnancy affects the female partner of a patient both data about the pregnancy and data about the treatment of the patient are regarded.

In any case the form for Reporting of an SAE is to be filled out and to be faxed to the following addresses within 24 hours after knowledge:

KOMPETENZNETZ HEPATITIS
Study House
Carl-Neuberg-Strasse 1
30625 Hannover
Fax: +49(0)511-532 6820

and

MSD Sharp & Dohme GmbH
Arzneimittelsicherheit
Thomas – Dehler – Str. 27
81737 München
Fax: +49(0)89 - 4561-1352

The **death** of any subject during the study or within 30 days of study completion (as defined above), regardless of the cause, must be reported to the sponsor Hep-Net and MSD Sharp & Dohme GmbH, Munich, Germany within 24 hours of first becoming aware of the death.
If an autopsy is performed, the pseudonymised report must be provided to the sponsor.

7.5 Management of adverse events

Management of AEs is generally achieved by dose reduction (see Table 4); however, in the case of life-threatening adverse events, identification of cardiac disease or development of cardiac dysfunction, pregnancy, failure to comply with the requirement for the practice of birth control for both male and female patients, both PegIntron® and Rebetol® therapy should be discontinued permanently.

Upon complete recovery of the adverse event, treatment with interferon alfa-2b and ribavirin may be resumed at a dose that is 50% lower than the dose associated with the adverse event. If this dose is tolerated for at least two weeks, treatment may be resumed at the dose associated with the adverse event. If the adverse event recurs, a reduced, tolerated dose may be administered and maintained or the subject may be taken off the treatment optimization study at the physician's discretion. If a dose reduction is necessary because of an adverse event, the patient must be examined every 2 weeks.

General Remarks

Unscheduled visits may take place for evaluation of laboratory parameters relevant to clinical safety. An unscheduled visit to monitor hemoglobin will be performed within one week of the patient's last visit if the patient displays a hemoglobin decline which meets all of the following three criteria:

⇒ Decline in hemoglobin since the previous visit
⇒ Decline in hemoglobin by more than 2 g/dl since the baseline evaluation
⇒ Decline in hemoglobin below 11 g/dl.

At the physician's discretion, the patient or a household member will be trained in the subcutaneous injection technique.

Table 4: Rules for dose reductions of PegIntron® and Rebetol®

	Dose reduction	Permanent discontinuation of treatment
HEMOGLOBIN	< 10 g/dl (Rebetol®)	< 8.5 g/dl (Rebetol®)
WHITE BLOOD CELL (WBC)	< 1.5 X 10^9/l (PegIntron®)	< 1.0 X 10^9/l (PegIntron®)
GRANULOCYTE	< 0.75 X 10^9/l (PegIntron®)	< 0.5 X 10^9/l (PegIntron®)
PLATELET	< 50 X 10^9/l (PegIntron®)	< 30 X 10^9/l (PegIntron®)
CREATININE	n/a	> 3.0 mg/dl
ALT/AST	n/a	2 x baseline and >10 x ULN
BILIRUBIN-INDIRECT	> 5 mg/dl (or > 85.5 µmol/l)* (Rebetol®)	> 4mg/dl (or >68.4 umol/l) (for > 4 weeks)
BILIRUBIN-DIRECT		> 2.5 x ULN

* Discontinue ribavirin alone for at least 1 week and no more than 2 weeks. Also take a blood sample to determine clotting. If indirect bilirubin falls to < 2.5 mg/dl after ribavirin treatment is interrupted, ribavirin treatment may be restarted at the nearest reduced daily dose stated in the protocol. If bilirubin remains stable at values below 2.5 mg/dl for a period of 4 weeks, ribavirin treatment may be resumed at the full (100%) protocol dose.

The daily dose is reduced if indirect bilirubin again rises above 4 mg/dl. This dose is then maintained. Treatment may be continued only if indirect bilirubin remains below 2.5 mg/dl. If indirect bilirubin remains high (> 4 mg/dl for more than 4 weeks), PEG-interferon alfa-2b and ribavirin must be permanently discontinued.

If an adverse laboratory event persists that is not severe enough to mandate permanent discontinuation of the drugs, the reduced dose of PEG-interferon alfa-2b or ribavirin (whichever was reduced) may be maintained. If the ribavirin dose was reduced following a decline in hemoglobin to below 10 g/dl, the dose reduction must continue to apply for at least 4 weeks.

The reduced doses may also be maintained if the hemoglobin value is ≥ 12 g/dl for women and ≥ 13 g/dl for men. If the adverse laboratory event recurs, a reduced, previously tolerated dose may be administered and maintained or the subject may be taken off the treatment optimization study at the physician's discretion.

The dose reduction takes place by lowering the PEG-interferon alfa-2b injection volume and number of ribavirin capsules as described in the tables below. Subjects who develop life-threatening adverse events must have PEG-interferon alfa-2b and ribavirin discontinued definitely.

To ensure that daily dosing is maintained, the dose should be reduced, not the frequency of use.

Table 5: Reduction of Rebetol®

Initial ribavirin dose	Total number of capsules per day	Reduced dose	Reduced number of capsules per day
1400 mg/day	3-0-4	800 mg/day	2-0-2
1200 mg/day	3-0-3	800 mg/day	2-0-2
1000 mg/day	2-0-3	800 mg/day	2-0-2
800 mg/day	2-0-2	600 mg/day	1-0-2

Table 6: Reduction of PegIntron®

Current dose	Next dose reduction
150 µg	120 µg
135 µg, 120 µg	100 µg
105 µg, 100 µg	80 µg
90 µg, 80 µg	50 µg
70 µg, 60 µg	30 µg

7.6 Benefit-Risk-Assessment

The optimal treatment duration for patients without RVR is not known until now. While some patients may be treated for a shorter period of time to reduce costs and side-effects, others may need longer treatment to improve the response rates. In this study we intend to treat patients with chronic hepatitis C of genotype 2 or 3 having characteristics associated with poor treatment response for additional 12 or 24 weeks beyond the standard treatment of PEG-IFN alpha-2b plus ribavirin to obtain data for a possible therapy-optimisation. Patients may profit of a prolonged treatment i.e. higher chances for permanent cure. Since not all participating patients will benefit from the therapy immediately, a direct benefit may not be seen for each individual (see Chapter 1). No evidence is available at the time of the completion of the study protocol to the Hep-Net Study Group indicating that a prolonged

intake of the study medication may need special warnings or precautions, other than those noted in the SmPC. Since all participating patients will have to be treated with the study medication for 24 weeks prior to entering the study, the individual adverse drug reaction profile will be well known to the patient and investigator.

Possible risks of PegIntron® include flu-like symptoms, fever, chills, fatigue and malaise two to eight hours after the initial dose. The initial reactions are usually mild to moderate. Tachyphylaxis may occur after 3-5 doses. Patients may take acetaminophen one hour before administration of PegIntron® and every 4 hours thereafter until the flu-like symptoms resolve. Changes of blood-picture are well known (see above) as well as a depression may occur. Further details are described in the SmPC.

Possible risks of Ribavirin® include changes of the blood-picture, especially anaemia (see above). Also itching and rashes are well known. Rare cases include insomnia and cough and increased levels of uric acid.
Animal experiments showed that administration of ribavirin to pregnant experimental animals sometimes resulted in the death of an embryo or caused congenital abnormalities in some of the offspring. There is no clinical data to demonstrate potentially harmful effects on unborn children, but it is assumed that the effects in human subjects would be similar to those seen in animal experiments. Animal studies also show that sperm morphology (structure) changes during ribavirin treatment. Ribavirin may persist in sperm fluid for two sperm generations. Therefore, men must also practice contraception during treatment and for 7 months after discontinuing ribavirin. Further details are described in the SmPC.

The fatigue typical of the first few weeks of interferon treatment may be more frequent or severe when ribavirin is added to the PEG-Interferon alfa-2b regimen. Therefore, patients on this combination should exercise caution and avoid driving motor vehicles or operating machinery, or consult their physician before driving a car or other vehicles or operating power tools or machinery.

8 STATISTICAL METHODS AND SAMPLE SIZE

8.1 Statistical Methods

The primary statistical analysis will be the comparison of rates for SVR in the ITT population (all randomized patients who received at least one PEG-Interferon injection) in the group with a prolongation of treatment by 24 weeks and a total treatment duration of 48 weeks (Group A) in comparison with the SVR rates in patients without treatment prolongation. The SVR rates of the latter group are assumed to be π_0=70% from historical data. According to the study design and the main objective, a one-sided test for proportions with a significance level of α=5% will be used to test the following hypothesis:

H_0: $\pi_0 = \pi_{24\ ext}$ versus H_1: $\pi_0 \leq \pi_{24\ ext}$

As main secondary statistical analysis, an analogous test is performed for Group B (treatment prolongation by 12 weeks for a total treatment duration of 36 weeks), again with a significance level of α=5%.

H_0: $\pi_0 = \pi_{12\ ext}$ versus H_1: $\pi_0 \leq \pi_{12\ ext}$

As further secondary statistical analysis, a comparison of SVR rates of Group A and Group B is planned, testing the one-sided hypothesis

H_0: $\pi_{12\ ext} = \pi_{24\ ext}$ versus H_1: $\pi_{12\ ext} \leq \pi_{24\ ext}$.

Further explorative analysis are planned to compare clinical variables and secondary end points between Group A (48 weeks) and B (36 weeks) with appropriate statistical methods as, e.g., Fishers exact test, χ^2 test, t-test, Mann-Whitney U test and corresponding confidence intervals will be used. Typically, tests will use a significance level of alpha=5% and can be one- or two-sided. Further explorative statistical analysis, e.g., the analysis of predictors for SVR may be used if appropriate.

8.2 Sample Size Justification

The primary end point is the rate π of SVR. The main objective of the study is to compare the SVR rates of a treatment extension of 24 or 12 weeks in comparison with π_0=70 %, the rate which can be assumed for SVR rates without treatment prolongation from historical data of 24 weeks overall treatment duration.

According to the study design and the main objective, a one-sided test for proportions with a significance level of α=5% will be used. The primary statistical analysis will use an ITT approach (all randomized patients who received at least one PEG-Interferon injection). Sample size is chosen such that not only the primary statistical analysis but also the main secondary statistical analysis has a high power and that a comparison of both treatment groups which will be done as further secondary statistical analysis still has a power above 50%.

Power calculation is based on assuming an improved rate of SVR of $\pi_{24\ ext}$= 95% for Group A and of $\pi_{12\ ext}$=85% for Group B which will be compared with the rate of SVR of π_0=70%, respectively. Then we obtain a power of at least 90 %, if ITT information of at least 75 patients in Group B is available (main secondary statistical analysis) and a power of nearly 100% if ITT information of at least 75 patients in Group A (primary statistical analysis), is available. The power to prove a statistical difference between Group A and B (further secondary analysis) with a one-sided test and α=5% is 55% (using continuity correction).

8.3 Historical control group

Ref.	Treatment regimen	Patients without RVR	SVR without RVR
21	1,5 µg/kg 24 weeks PEG-IFN alpha-2b plus 800-1400 mg ribavirin (24 weeks)	HCV-genotype 2 (n=0)<br>HCV genotype 3 (n=35)	n.a.<br>24/35* (68.6%)
7	1,5 µg/kg 24 weeks PEG-IFN alpha-2b plus 800-1400 mg ribavirin (24 weeks)	HCV-genotype 2 (n=20)<br>HCV genotype 3 (n=96)	15/20** (75%)<br>54/96** (56.3%)
8, 21	1,5 µg/kg 24 weeks PEG-IFN alpha-2b plus 800-1400 mg ribavirin (24 weeks)	HCV-genotype 2/3 (n=151)	93/151 (61,6%)

* Patients who were HCV-RNA positive (>29 IU/mL) at week 4 but HCV-RNA negative at week 12
** Patients who were HCV-RNA positive (>50 IU/mL) at week 4 but HCV-RNA negative at week 12

Ref.	Treatment regimen	Patient groups	SVR without RVR
21	1,5 µg/kg 24 weeks PEG-IFN alpha-2b plus 800-1400 mg ribavirin (24 weeks)	HCV-genotype 2 (n=42)<br>HCV genotype 3 (n=182)	n.a.<br>24/35* (68.6%)
6	1,5 µg/kg 24 weeks PEG-IFN alpha-2b plus 800-1400 mg ribavirin (24 weeks)	HCV-genotype 2 (n=51)<br>HCV genotype 3 (n=211)	15/20** (75%)<br>54/96** (56.3%)

* Patients who were HCV-RNA positive (>29 IU/mL) at week 4 but HCV-RNA negative at week 12
** Patients who were HCV-RNA positive (>50 IU/mL) at week 4 but HCV-RNA negative at week 12

9 DATA QUALITY ASSURANCE

Each involved investigator is member (regular or associate) of Hep-Net and experienced in Interferon-based therapy of patients with chronic viral hepatitis. As members of Hep-Net, the investigators take part in advanced trainings organized by the sponsor Hep-Net, regularly.
The data collected will be entered into an electronic CRF. A comprehensive validation check program will verify the data and discrepancy reports (Data Queries) will be generated accordingly for resolution by the investigator. Accurate and reliable data collection will be assured by verification and cross-check of the electronic CRFs against the investigator's records by the study monitor (source document verification), and the maintenance of a drug-dispensing log by the investigator.

10 ETHICAL ASPECTS

10.1 Local Regulations/Declaration of Helsinki

The sponsor will ensure that this study fully adheres to the principles outlined in the "Guideline for Good Clinical Practice" which has its basis in the principles of the "Declaration of Helsinki" and with the laws and regulations of the country in which the research is conducted, whichever affords the greater protection of the individual.

10.2 Patient Information and Informed Consent

It is the responsibility of the investigator or an authorised physician to obtain written informed consent from each individual participating in this study after adequate explanation of the aims, methods, objectives and potential hazards of the study. It must also be explained to the patients that they are completely free to refuse to enter the study or to withdraw from it at any time for any reason without any consequences for further treatment. Appropriate forms for obtaining written informed consent will be provided to the investigator by Hep-Net/designee.

If new safety information results in significant changes in the risk/benefit assessment, the consent form should be reviewed and updated if necessary. All patients (including those already being treated) should be informed of the new information, given a copy of the revised form and asked to give their consent to continue in the study.

10.3 Insurance

Every patient participating in the study will be insured by ALLIANZ AG according to German Arzneimittelgesetz (AMG) § 40 Abs. 3. All subjects will be informed about their rights and obligations in regard to insurance policies before participating in the study. A copy of the insurance policies will be available for each subject.

10.4 Independent Ethics Committees (IEC)

This protocol and any accompanying material provided to the patient (such as patient information sheets or informed consent) as well as any advertising will be submitted by the sponsor to an Independent Ethics Committee (IEC). Approval from the committee must be obtained before starting the study.

Any substantial modifications made to the protocol after receipt or the IEC approval must also be submitted to the Committee in accordance with local procedures and regulatory requirements.

10.5 Financing

The study will be financed by the Bundesministerium für Bildung und Forschung (BMBF)-Project German Competence Network for Viral Hepatitis (Hep-Net). Study medication will be provided by MSD Sharp & Dohme GmbH.
No compensation will be given to the patients for participating in this study. Only travel expenses will be reimbursed for enrolled patients.

11 CONDITIONS FOR MODIFYING THE PROTOCOL

No changes to the study protocol will be allowed unless discussed in detail with the Hep-Net Study coordinators and filed as an amendment/modification to this protocol.
Any amendment/modification to the protocol will be adhered to by the participating centre(s) and will apply to all subjects. Written IEC approval of protocol amendments is required for substantial amendments prior to implementation; modifications are submitted to IECs for information only.

12 SUBJECT COMPLETION

After finishing the study, patients will be treated according to standard therapeutic procedures applicable at that time.

12.1 Procedures for handling patients lost to follow-up and drop-outs

Investigators should make an attempt to contact those patients who do not return for scheduled visits or follow-up. Information gathered should be described on the Study Conclusion page of the Case Report Form and on Medication/Adverse event forms.

12.2 Reasons for drop-out

It should be specified on the Study Conclusion page of the case report form which of the following possible reasons were responsible for drop-out of the patient from the study:

- Serious adverse event
- Non-serious adverse event
- Protocol violation (specify)
- Consent withdrawal, not due to an adverse event
- Migration from the study area
- Lost to follow-up
- Other (specify)

13 STUDY DOCUMENTATION, electronic CRF AND RECORD KEEPING

13.1 Investigator's Files/Retention of Documents and Source Documents

The Investigator must maintain adequate and accurate records to enable the conduct of the study to be fully documented and the study data to be subsequently verified. These documents should be classified into two different separate categories (1) Trial Investigator's File (TIF), and (2) patient clinical source documents.
The TIF will contain the protocol/amendments, Independent Ethics Committee and governmental approval with correspondence, sample patient information and informed consent form, general insurance conditions, signed informed consent forms from enrolled patients, drug records, patient screening and enrolment logs, signed staff curriculum vitae and authorization forms and other appropriate documents/correspondence etc.

Patient clinical source documents (usually defined by the project in advance to record key efficacy/safety parameters) would include patient hospital/clinic records, physician's and nurse's notes, original laboratory reports, ECG, and special assessment reports, consultant letters.
The investigator must keep these two categories of documents on file for at least 15 years after completion or discontinuation of the study. After that period of time the documents may be destroyed, subject to local regulations.
Should the Investigator wish to assign the study records to another party or move them to another location, Hep-Net must be notified in advance.
If the Investigator can not guarantee this archiving requirement at the investigational site for any or all of the documents, special arrangements must be made between the Investigator and Hep-Net to store these in a sealed container(s) outside of the site so that they can be returned sealed to the Investigator in case of a regulatory inspection. Where source documents are required for the continued care of the patient, appropriate copies should be made for storing outside of the site.

The investigator shall supply the sponsor/designee on request with any required background data from the study documentation or clinic records. This is particularly important for quality assurance (monitoring of source documents, audits), when errors in data transcription are suspected and in case of requests for inspections.

13.2 Audits and Inspections

The Investigator should understand that source documents for this study should be made available to appropriately qualified personnel from the Hep-Net Quality Assurance Unit or its designees or to health authority inspectors after appropriate notification.

13.3 Case Report Forms (CRFs)

For each patient enrolled, an electronic CRF must be completed promptly after the patients visit and signed by the (principal) investigator. This also applies to records for those patients who fail to complete the study. If a patient withdraws from the study, the reason must be noted on the CRF. If a patient is withdrawn from the study because of a treatment-limiting adverse event, thorough efforts should be made to clearly document the outcome.
All forms should be typed or filled out using indelible ink, and must be legible. Errors should be crossed out but not obliterated, the correction inserted, and the change initialled and dated by the investigator or his/her authorized delegate. The investigator should ensure the accuracy, completeness, legibility, and timeless of the data reported to the sponsor in the CRFs and all required reports.

14 MONITORING THE STUDY

Monitoring of the study is required according to ICH-GCP and AMG and is important to guarantee a valid study.

14.1 Direct Source Data Verification

On-Site-monitoring: 10 % of all filled-in case report forms in each study site will undergo a 100 %-verification to the source of data. Further case report forms will only be checked with respect to key data, e.g. informed consent and inclusion/exclusion criteria. Further details will be described in a trial specific monitoring plan.

15 CONFIDENTIALITY OF TRIAL DOCUMENTS AND PATIENTS RECORDS

The investigator must assure that patients` anonymity will be maintained and that their identities are protected from unauthorized parties. The investigator should keep a patient enrolment log relating codes to the names of patients. The investigator should maintain documents not for submission to Hep-Net, e.g., patients written consent forms, in strict confidence.

16 PUBLICATION OF DATA

The results of the study shall be published in an international peer-reviewed journal. The publication rules of the standing orders of the German Competence Network for Viral Hepatitis (Hep-Net) shall be applied.
The trial will be registered at a study register (i.e. www.clinicaltrials.gov) according to current publication guidelines.

17 SIGNATURES

The following persons agree with the content of the clinical study and confirm the protocol in the latest version.

Sponsor
Competence Network Viral Hepatitis (Hep-Net), Prof. Dr. M. P. Manns, M.D., Germany

Date: ________________________ Signature.__________________________

Coordinating Investigator
Prof. Dr. med. M.P. Manns, Hannover Medical School, Germany

Date:________________________ Signature.__________________________

Investigator/Study Center

Name (print):____

Study Center: ____

Date: ________________________ Signature.__________________________

18 REFERENCE LIST

1. **Aghemo, A., M. G. Rumi, R. Soffredini, R. D'Ambrosio, G. Ronchi, N. E. Del, S. Gallus, and M. Colombo**. 2006. Impaired response to interferon-alpha2b plus ribavirin in cirrhotic patients with genotype 3a hepatitis C virus infection. Antivir. Ther. **11**:797-802.

2. **Choo, Q. L., G. Kuo, A. J. Weiner, L. R. Overby, D. W. Bradley, and M. Houghton**. 1989. Isolation of a cDNA clone derived from a blood-borne non-A, non-B viral hepatitis genome. Science **244**:359-362.

3. **Cornberg, M., K. Deterding, and M. P. Manns**. 2006. Present and future therapy for hepatitis C virus. Expert. Rev. Anti. Infect. Ther. **4**:781-793.

4. **Cornberg, M., H. Wedemeyer, and M. P. Manns**. 2002. Treatment of chronic hepatitis C with PEGylated interferon and ribavirin. Curr. Gastroenterol. Rep. **4**:23-30.

5. **Dalgard, O., K. Bjoro, K. B. Hellum, B. Myrvang, S. Ritland, K. Skaug, N. Raknerud, and H. Bell**. 2004. Treatment with pegylated interferon and ribavarin in HCV infection with genotype 2 or 3 for 14 weeks: a pilot study. Hepatology **40**:1260-1265.

6. **Dalgard, O., K. Bjoro, H. Ring-Larsen, E. Bjornsson, M. Holberg-Petersen, E. Skovlund, O. Reichard, B. Myrvang, B. Sundelof, S. Ritland, K. Hellum, A. Fryden, J. Florholmen, and H. Verbaan**. 2008. Pegylated interferon alfa and ribavirin for 14 versus 24 weeks in patients with hepatitis C virus genotype 2 or 3 and rapid virological response. Hepatology **47**:35-42.

7. **Dalgard, O., K. Bjoro, H. Ring-Larsen, E. Bjornsson, M. Holberg-Petersen, E. Skovlund, O. Reichard, B. Myrvang, B. Sundelof, S. Ritland, K. Hellum, A. Fryden, J. Florholmen, and H. Verbaan**. 2008. Pegylated interferon alfa and ribavirin for 14 versus 24 weeks in patients with hepatitis C virus genotype 2 or 3 and rapid virological response. Hepatology **47**:35-42.

8. **Dalgard, O., K. Bjoro, H. Ring-Larsen, E. Bjornsson, M. Holberg-Petersen, E. Skovlund, O. Reichard, B. Myrvang, B. Sundelof, S. Ritland, K. Hellum, A. Fryden, J. Florholmen, and H. Verbaan**. 2008. Pegylated interferon alfa and ribavirin for 14 versus 24 weeks in patients with hepatitis C virus genotype 2 or 3 and rapid virological response. Hepatology **47**:35-42.

9. **Davis, G. L., J. E. Albright, S. F. Cook, and D. M. Rosenberg**. 2003. Projecting future complications of chronic hepatitis C in the United States. Liver Transpl. **9**:331-338.

10. **Hoofnagle, J. H.** 1997. Hepatitis C: the clinical spectrum of disease. Hepatology **26**:15S-20S.

11. **Hoofnagle, J. H.** 2002. Course and outcome of hepatitis C. Hepatology **36**:S21-S29.

12. **Hoofnagle, J. H., K. D. Mullen, D. B. Jones, V. Rustgi, A. Di Bisceglie, M. Peters, J. G. Waggoner, Y. Park, and E. A. Jones**. 1986. Treatment of chronic non-A,non-B

hepatitis with recombinant human alpha interferon. A preliminary report. N. Engl. J. Med. **315**:1575-1578.

13. **Lauer, G. M. and B. D. Walker**. 2001. Hepatitis C virus infection. N. Engl. J. Med. **345**:41-52.

14. **Mangia, A., R. Santoro, N. Minerva, G. L. Ricci, V. Carretta, M. Persico, F. Vinelli, G. Scotto, D. Bacca, M. Annese, M. Romano, F. Zechini, F. Sogari, F. Spirito, and A. Andriulli**. 2005. Peginterferon alfa-2b and ribavirin for 12 vs. 24 weeks in HCV genotype 2 or 3. N. Engl. J. Med. **352**:2609-2617.

15. **Manns, M. P., J. G. McHutchison, S. C. Gordon, V. K. Rustgi, M. Shiffman, R. Reindollar, Z. D. Goodman, K. Koury, M. Ling, and J. K. Albrecht**. 2001. Peginterferon alfa-2b plus ribavirin compared with interferon alfa-2b plus ribavirin for initial treatment of chronic hepatitis C: a randomised trial. Lancet **358**:958-965.

16. **Manns, M. P. and E. G. Rambusch**. 1999. Autoimmunity and extrahepatic manifestations in hepatitis C virus infection. J. Hepatol. **31 Suppl 1**:39-42.

17. **Manns, M. P., H. Wedemeyer, and M. Cornberg**. 2006. Treating viral hepatitis C: efficacy, side effects, and complications. Gut **55**:1350-1359.

18. **Shepard, C. W., L. Finelli, and M. Alter**. 2005. Global epidemiology of hepatitis C virus infection. Lancet Infectious Diseases **5**:558-567.

19. **Shiffman, M. L., F. Suter, B. R. Bacon, D. Nelson, H. Harley, R. Sola, S. D. Shafran, K. Barange, A. Lin, A. Soman, and S. Zeuzem**. 2007. Peginterferon alfa-2a and ribavirin for 16 or 24 weeks in HCV genotype 2 or 3. N. Engl. J. Med. **357**:124-134.

20. **von Wagner, M., M. Huber, T. Berg, H. Hinrichsen, J. Rasenack, T. Heintges, A. Bergk, C. Bernsmeier, D. Haussinger, E. Herrmann, and S. Zeuzem**. 2005. Peginterferon-alpha-2a (40KD) and ribavirin for 16 or 24 weeks in patients with genotype 2 or 3 chronic hepatitis C. Gastroenterology **129**:522-527.

21. **Zeuzem, S., R. Hultcrantz, M. Bourliere, T. Goeser, P. Marcellin, J. Sanchez-Tapias, C. Sarrazin, J. Harvey, C. Brass, and J. Albrecht**. 2004. Peginterferon alfa-2b plus ribavirin for treatment of chronic hepatitis C in previously untreated patients infected with HCV genotypes 2 or 3. J. Hepatol. **40**:993-999.

19. STUDY SET-UP

Study monitoring and organisation:	Hannover Clinical Trial Center GmbH<br>Sabine Konopka, Armin Papkalla Ph.D,<br>Project Manager<br>Carl-Neuberg-Strasse 1 / K27<br>D-30625 Hannover<br>Telephone: +49 511 53 33 33 0<br>Fax: +49 511 53 33 33 99
Central Lab for HCV-RNA analysis:	Hannover Medical School,<br>Clinic for Gastroenterology, Hepatology, and Endocrinology,<br>Prof. Manns, Dr. H. Wedemeyer<br>Carl-Neubergstr.1<br>D-30625 Hannover<br>Telephone: +49 511 532 6814
